# ﻿Diversity and host plant utilization of leaf-mining beetles of Chrysomeloidea (Coleoptera) in Japan

**DOI:** 10.3897/zookeys.1238.124514

**Published:** 2025-05-16

**Authors:** Makoto Kato, Yume Imada

**Affiliations:** 1 Graduate School of Human and Environmental Studies, Kyoto University, Sakyo, Kyoto, 606-8501, Japan Kyoto University Kyoto Japan; 2 Graduate School of Science, Kyoto University, Kitashirakawa Oiwake-cho, Sakyo, Kyoto, 606-8502, Japan Kyoto University Kyoto Japan

**Keywords:** Cerambycidae, Chrysomelidae, host specificity, leaf miner, Megalopodidae, mining pattern

## Abstract

The superfamily Chrysomeloidea (Cerambycidae + Chrysomelidae + Megalopodidae) encompasses a diverse phytophagous beetles, whose larvae exhibit internal or external feeding on leaves, wood, or roots of many plants. Through extensive research on leaf-mining insects in Japan, 64 species of Chrysomeloidea were confirmed to engage in leaf-mining behavior during their larval stages infesting tracheophytes, and comprising 2 Cerambycidae, 9 Megalopodidae, and 53 Chrysomelidae. This study presents an overview of the host plants and mining patterns of these 64 leaf-mining beetle species and describes two new species, *Sphaerodermakomiana* Kato, **sp. nov.** and *Dactylispaadinae* Kato, **sp. nov.** The leaf-mining beetles demonstrate a broad host range including Equisetales, Polypodiales, Cycadales, and 23 orders of angiosperms. Particularly notable diversification was observed on Polypodiales (within *Halticorcus*), Ranunculaceae (*Argopus* and *Sphaeroderma*), Celastraceae (*Zeugophora*), and Oleaceae (*Argopistes*). Host specificity greatly varied among the reported 64 beetle species: 29 spp. species-specific; 12 spp. genus-specific; 16 spp. family-specific; 2 spp. order-specific; 5 spp. non-specific even at order level. The five non-specific species (*Argopuspunctipennis*, *Sphaerodermanigricolle*, *Dactylispaangulosa*, *Notosacanthaihai*, and *N.loochooana*) are associated with multiple plant orders while maintaining specificity to a small number of genera belonging to phylogenetically distant plant families. This pattern, termed as extended host specificity, suggests recent host shifts across plant families without substantial expansion of host ranges.

## ﻿Introduction

Leaf mining refers to the lifestyle of phytophagous insect larvae that live, feed, and develop within the internal tissues of plant leaves. This behavior occurs in four holometabolous insect orders: Coleoptera, Hymenoptera, Diptera, and Lepidoptera (Hering 1951). The earliest credible fossil records of leaf mines date back to the Late Triassic period (Imada et al. 2022) and even to the Late Carboniferous period (Knecht et al. 2023). Leaf-mining insects typically exhibit dorso-ventrally flattened bodies and well-developed mouthparts adaptations for living within the confined spaces of leaves and consuming fibrous plant tissues (Hering 1951). While the plant tissue offers internal leaf miners protection from insect and vertebrate predators, as well as environmental stress, these larvae are still vulnerable to parasitoid attacks. This ecological niche has been adopted by diverse insect leaf-miners.

Leaf-mining species are represented in several coleopteran families, including the Buprestidae, Nitidulidae, Mordellidae, Megalopodidae, Chrysomelidae, Attelabiidae, Brachyceridae, and Curculionidae (Hering 1951; Tooker and Giron 2020; Eiseman 2022). The latter five are part of the clade Phytophaga, which spans two superfamilies, Chrysomeloidea and Curculionoidea, and evolved from the fungus-feeding beetle superfamily Cucujoidea (Hunt et al. 2007; Zhang et al. 2018-. These superfamilies, comprising 59,000 and 53,000 species, respectively, have diversified enormously as angiosperm plants radiated since the Cretaceous period (Farrell 1998; Hunt et al. 2007). The Chrysomeloidea comprises three families: Cerambycidae, Megalopodidae, and Chrysomelidae. Cerambycids are typically wood or shoot borers during their larval stage, while megalopodid and chrysomelid larvae feed on leaves or roots, either internally or externally, of both terrestrial and aquatic plants. The oldest known Chrysomeloidea fossil, attributed to the Cerambycidae, was found in the Lower Cretaceous Yixian Formation of China (Wang et al. 2014).

Chrysomeloidea contains diverse leaf-mining species across more than 40 genera (Jolivet and Hawkeswood 1995; Santiago-Blay 2004; Eiseman 2022; Frost 1924). Within Megalopodidae, at least a few *Zeugophora* species are leaf miners (Takemoto 2019). The Chrysomelidae family exhibits larval habits that vary from leaf mining and root boring to external leaf feeding. Among its ten subfamilies (Reid 1995), both Galerucinae and Cassidinae harbor leaf-mining species (Cai et al. 2022). Within Galerucinae in Japan, leaf-mining species have been identified in several genera, including *Phyllotreta* Chevrolat, 1837, *Mantura* Stephens, 1831, *Halticorcus* Lea, 1917 (= *Schenklingia*), *Argopistes* Motschulsky, 1860, *Argopus* Fischer von Waldheim, 1824, and *Sphaeroderma* Stephens, 1831 (Yano 1954, 1958, 1965; Kato 1991; Kimoto and Takizawa 1993). The Hispini tribe within Cassidinae span tropical and subtropical regions and exhibit several leaf-mining species (Chaboo 2007). Japanese Hispini leaf-mining species are found in the genera *Leptispa* Baly, 1858, *Asamangulia* Maulik, 1915, *Dactylispa* Weise, 1897, *Hispellinus* Weise, 1897, *Rhadiosa* Weise, 1905, *Platypria* Guérin-Méneville, 1840, and *Dicladispa* Gestro, 1897 (Kimoto and Takizawa 1993). Additionally, in the Notosacanthini within the subfamily Cassidinae, *Notosacantha* Chevrolat, 1837 also includes leaf-mining species (Rane et al. 2000; Monteith et al. 2021). Despite the abundance of taxonomic studies of chrysomelids in these genera (Kimoto and Takizawa 1993; Takizawa 2005, 2007, 2015, 2021), reports on their larval biology remain limited. Leaf mines of chrysomelids have been documented in just 15 species in Japan, as reported by Yano (1954, 1965) and Kato (1991). A more comprehensive understanding of the association between leaf-mining chrysomelids and their host plants could provide insights into the diversification processes within the Chrysomeloidea.

During the last 40 years, we have conducted extensive collections of Japanese leaf miners, reared them, and collected data on their diversity, host plants, and larval biology. In this article, we present a detailed account of this leaf-mining beetle diversity, including the identification of two new chrysomelid species within the genera *Sphaeroderma* and *Dactylispa*. Additionally, we explore the host plants and host specificity of these leaf-mining species, with insights into leaf mines and their larval biology, and discuss the association between leaf-mining beetles and their host plants.

Our study reveals the presence of 64 leaf-mining species within Japanese Chrysomeloidea, each intricately associated with specific host plants. While most of these beetle species exhibit host specificity at the genus or species level, we have observed instances where certain species are associated with multiple genera belonging to phylogenetically distant plant families. By investigating whether these species demonstrate specialist or generalist tendencies, we have categorized the pattern of host selection as exhibiting extended host specificity.

## ﻿Materials and methods

Since the 1980s, MK has conducted extensive sampling of chrysomelid leaf mines across the Japanese Archipelago and all specimens were collected by MK, with specific exceptions noted. To rear the leaf-mining chrysomelid larvae, mined leaves were placed in plastic cases with a layer of vermiculite at the bottom. This setup was kept moderately moist to create an optimal environment for the pupation and hibernation phases usually in incubators. Approximately 400 adult chrysomelid beetles were obtained by rearing the leaf-mining larvae. Leaves with leaf mines were dried as herbarium specimens and deposited in the Kyoto University Museum, Japan (**KUM**). Plant species were identified according to their scientific names, in line with Ohashi et al. (2015). The insect specimens were also deposited in the KUM, with the type specimens deposited in National Museum of Nature and Science, Tokyo (**NSMT**). All material with a registration number is mentioned in the listing of the designated material type.

For morphological examination, adult specimens were examined under a microscope (VHS-7000; Keyence, Osaka, Japan). This analysis was augmented by synthesizing virtual images from a series of depth-focused photographs to capture detailed images of the specimens. For identification we referred to Hayashi et al. (1984), Kimoto and Takizawa (1993, 1997). To observe male and female genitalia, the abdomens were macerated in in a 10% KOH solution for ~ 12 h at room temperature, rinsed in water, and dissected under a microscope.

## ﻿Results

From the Japanese Archipelago, we confirmed 64 species of Chrysomeloidea as leaf miners, including two cerambycids, nine megalopodids, and 53 chrysomelids (Table 1). We provide a summary of host plants and leaf mines of these species and describe two new species belonging to *Sphaeroderma* and *Dactylispa*. The following list also includes ten beetle species that are unknown for their larval habits but are suspected to be leaf-miners in light of the habits of morphologically related species. In the following descriptions, families, subfamilies, tribes, and genera are arranged in a phylogenetic order suggested by Hayashi et al. (1984), and species in each genus are arranged in a taxonomic order of their host plants. In the genus *Dactylispa*, species are arranged by their species groups as suggested by Zhang et al. (2021).

**Table 1.** Leaf-mining species of Chrysomeloidea in Japan, with their host plants, host specificity, mining patterns and pupation sites.

Family	Subfamily	Tribe	Genus	Species	Host plants	No. utilized plant taxa				Mining pattern*	Ps**
						order	family	genur	species		
Cerambycidae	Lamiinae		*Mimectatina*	*meridianaohirai*	**Cycadaceae**: *Cycasrevoluta*	1	1	1	**1**	LBM	in
			*Sybra*	*ordinata*	**Cycadaceae**: *Cycasrevoluta*	1	1	1	**1**	LBM	in
Megalopodinae	Zeugophorinae		*Zeugophora*	*annulata*	**Celastraceae**: *Celastrusorbiculatus*, *Euonymusalatus*, *E.sieboldianus*, *E.oxyphyllus*, *E.macropterus*, *E.tricarpus*, *E.melananthus*, *E.japonicus*, *E.fortunei*, *Tripterygiumregelii*	1	**1**	3	10	LM	ex
				*chujoi*	**Celastraceae**: *Euonymusfortunei*	1	1	1	**1**	PM↓→LM	ex
				*flavonotata*	**Celastraceae**: *Euonymustanakae*, *E.japonicus*	1	1	**1**	2	LM	ex
				*nigricolis*	**Celastraceae**: *Euonymussieboldianus*	1	1	1	**1**	LBM	ex
				*unifasciata*	**Celastraceae**: *Euonymussieboldianus*	1	1	1	**1**	LM	ex
				*varipes*	**Symplocaceae**: *Symplocossawafutagi*, *S.coreana*	1	1	**1**	2	LBM	ex
				*hozumii*	**Salicaceae**: Salixcardiophyllavar.urbaniana	1	1	1	**1**	LBM	ex
				*japonica*	**Salicaceae**: *Populussuaveolens*	1	1	1	**1**	LBM	ex
				*cupka*	**Salicaceae**: *Populussuaveolens*†	1	1	1	**1**	?	ex
				*gracilis*	?					?	?
Chrysomelidae	Alticinae	Alticini	*Phyllotreta*	*ezoensis*	**Brassicaceae**: *Drabanemorosa*	1	1	1	**1**	PM⟷RM	ex
				*shirahatai*	**Brassicaceae**: *Cardamineleucantha*	1	1	1	**1**	LM	ex
			*Longitarsus*	*holsaticus*	**Plantaginaceae**: *Pennellianthusfrutescens*	1	1	1	**1**	RM	ex
			*Dibolia*	*japonica*	**Lamiaceae**: Stachysasperavar.hispidula	1	1	1	**1**	LBM	ex
			*Mantura*	*clavareaui*	**Polygonaceae**: *Rumexjaponicus*, *R.acetosa*, *Polygonumaviculare*	1	**1**	2	3	LBM	ex
				*fulvipes*	**Oxalidaceae**: *Oxaliscorniculata*	1	1	1	**1**	LBM	ex
				*japonica*	?					?	?
			*Hippuriphila*	*babai*	**Equisetaceae**: *Equisetumarvense*, *E.fluviatile*	1	1	**1**	2	PM	ex
			*Psylliodes*	*punctifrons*	**Brassicaceae**: *Brassicajuncea*, *Cardamineocculta*, *C.regeliana*, Raphanussativusvar.raphanistroides, *Rorippapalustris*, *Eutremajaponicum*	1	**1**	4	6	PM⟷RM	ex
				*subrugosa*	**Brassicaceae**: *Arabishirsuta*	1	1	1	**1**	PM⟷RM	ex
			*Halticorcus*	*kasuga*	**Polypodiaceae**: *Lepisorusthunbergianus*, *L.onoei*, *L.miyoshianus*	1	1	**1**	3	LBM	ex
				*sauteri*	**Dryopteridaceae**: *Crytomiumfalcantum*; Oleandraceae: *Nephrolepiscordifolia*; **Polypodiaceae**: *Colysiselliptica*, *Loxogrammesalicifolia*, *Leptochilusneopothifolius*, *Phymatosorusscolopendria*	**1**	3	6	6	LBM	ex
				*hiranoi*	**Aspleniaceae**: *Aspleniumantiquum*; **Polypodiaceae**: *Pyrrosialingua*, *Lemmaphyllummicrophyllum*, *Lepisorusthunbergianus*, *Loxogrammesalicifolia*; **Vittariaceae**: *Vittariaflexuosa*	**1**	3	6	6	LBM	ex
				*duodecimmaculata*	**Polypodiaceae**: *Phymatosorusscolopendria*, *Selligueayakushimensis*	1	**1**	2	2	LBM	ex
			*Argopistes*	*coccinelliformis*	**Oleaceae**: *Ligustrumjaponicum*, *L.micranthum*, *L.ovalifolium*, *Osmanthusheterophyllus*†, *O.zentaroanus*, *O.×fortunei*†	1	**1**	2	6	LBM	ex
				*biplagiata*	**Oleaceae**: *Ligustrumjaponicum*, *Osmanthusheterophyllus*, *O.zentaroanus* , *O.×fortunei*	1	**1**	2	3	LBM	ex
				*unicolor*	**Oleaceae**: *Osmanthusheterophyllus*†	1	1	1	**1**	LBM	ex
				*ryukyuensis*	**Oleaceae**: *Ligustrumjaponicum*	1	1	1	**1**	LBM	ex
				*tsekooni*	**Oleaceae**: *Ligustrumobtusifolium*, *Fraxinussieboldiana*, *Syringareticulata*	1	**1**	3	3	LBM	ex
			*Argopus*	*balyi*	**Ranunculaceae**: *Clematisstans*, *C.apiifolia*†, *Clematisterniflora*†	1	1	**1**	2	LBM	ex
				*clypeatus*	**Ranunculaceae**: *Clematisterniflora*, *C.apiifolia*†	1	1	**1**	2	LBM	ex
				*punctipennis*	**Aristolochiaceae**: *Asarumheterotropoides*, *A.tohokuense*, *A.sieboldii*, *A.caulescens*, *A.megacalyx*, *A.blumei*, *A.curvistigma*, *A.asperum*, *A.nipponicum*; **Ranunculaceae**: *Aconitumpterocaule*, *A.gigas*, *A.sachalinense*, *A.japonicum*, *A.okuyamae*; **Asteraceae**: *Cirsiumjaponicum*, *C.kiotoense*, *C.yoshinoi*, *C.taishakuense*, *C.otayae*, *C.microspicatum*, *C.ugoense*, *C.otayae*, *C.makinoi*, *C.kamtschaticum*, *C.austrohidakaense*	**1**	3	3	25	LBM	ex
				*clarki*	?					?	?
				*nigripennis*	?					?	?
				*unicolor*	?					?	?
			*Sphaeroderma*	*nigricolle*	**Liliaceae**: *Cardiocrinumcordatum*, *Liliumauratum*, *Tricyrtismacropoda*, *T.flava*, Smilacaceae: *Smilaxchina*, *Smilaxriparia*, *S.nipponica*, *S.stans*, *Heterosmilaxjaponica*; **Stemonaceae**: *Croomiaheterosepala*, *C.japonica*	**1**	3	6	11	LBM	ex
				*japanum*	**Commelinaceae**: *Commelinacommunis*	1	1	1	**1**	LBM	ex
				*tarsatum*	**Poaceae**: *Phyllostachysbambusoides*, *P.bambusoides*, Pleioblastuschinovar.viridis, *Sasakurilensis*, *S.senanensis*, *S.nipponica*, *Sasamorphaborealis*, *Shibataeakumasaca*, Stipacoreanavar.kengii	1	**1**	6	9	LBM	ex
				*seriatum*	**Poaceae**: *Panicumbisulcatum*	1	1	1	**1**	LBM	ex
				*apicale*	**Poaceae**: *Miscanthussinensis*†	1	1	1	**1**	LBM	ex
				*fulvoapicale*	?					?	?
				*akebia*	**Lardizabalaceae**: *Akebiatrifoliata*, *A.quinata*	1	1	**1**	2	BM	ex
				*inaizumii*	**Lardizabalaceae**: *Akebiatrifoliata*, *A.quinata*	1	1	**1**	2	BM	ex
				*quadrimaculatum*	**Ranunculaceae**: Clematistaiwanianavar.ryukiuensis	1	1	1	**1**	LBM	ex
				*flavonotatum*	**Ranunculaceae**: *Clematispierotii*	1	1	1	**1**	LBM	ex
				*placidum*	**Ranunculaceae**: *Clematisapiifolia*	1	1	1	**1**	BM	ex
				*unicolor*	**Ranunculaceae**: *Clematisterniflora*, *C.apiifolia*	1	1	**1**	2	LM	ex
				*uenoi*	**Ranunculaceae**: *Clematisapiifolia*, *C.terniflora*	1	1	**1**	2	LBM	ex
				*ohkuboi*	**Ranunculaceae**: *Cimicifugajaponica*, *C.biternata*, *C.simplex*	1	1	**1**	3	LBM	ex
				*separatum*	**Ranunculaceae**: *Clematisapiifolia*†	1	1	1	**1**	LBM	ex
				*balyi*	**Asteraceae**: *Farfugiumjaponicum*, *Paraseneciokamtschaticus*, *P.amagiensis*, *P.yatabei*, *Petasitesjaponicus*	1	**1**	3	5	LBM	ex
				*komiana* sp. nov.	**Asteraceae**: *Ixerisjaponica*, *Lactucaindica*, *Youngiajaponica*	1	**1**	3	3	LBM	ex
				*atrum*	?					?	?
				*morimotoi*	?					?	?
				*nigripes*	?					?	?
				*obscurum*	?					?	?
				*rubidum*	?					?	?
	Cassidinae	Hispini	*Leptispa*	*taguchii*	**Poaceae**: *Miscanthussinensis*†	1	1	1	**1**	LM	in
				*miyamotoi*	**Poaceae**: *Miscanthussinensis*†, *Saccharumofficinarum*†	1	**1**	2	2	LM	in
			*Asamangulia*	*yonakuni*	**Poaceae**: *Oryzasativa*†, *Miscanthussinensis*†	1	**1**	2	2	LBM	in
			*Dactylispa*	*subquadrata*	**Fagaceae**: *Castaneacrenata*, *Castanopsissieboldii*, *Quercusdentata*, *Q.aliena*, *Q.serrata*, *Q.variabilis*, *Q.glauca*	1	**1**	3	7	LBM	in
				*masonii*	**Asteraceae**: *Ainsliaeaacerifolia*, *Asteryomena*, Cacaliaauriculatavar.kamtschatica, Cirsiummicrospicatumvar.kiotoense, *C.ashiuense*, *C.longepedunculatum*, *C.inundatum*, *C.tonense*, *C.olygophyllum*, *C.microspicatum*, *C.okamotoi*, *C.confertissimum*, *C.suzukaense*, *C.sieboldii*, C.tashiroivar.hidaense, *Ligulariafischeri*, *L.hodgsonii*, Paraseneciohastatusssp.orientalis, P.farfarifoliusvar.bulbiferus, *Petasitesjaponicus*, *Syneilesispalmata*	1	**1**	8	21	LBM	in
				*angulosa*	**Lamiaceae**: Clinopodiumchinensesubsp.grandiflorum, C.micranthumvar.sachalinense, *C.gracile*, *Isodoninflexa*, *Glechomahederacea*, *Prunellavulgaris*, *Salviajaponica*, *S.ranzaniana*, *S.glabrescens*; **Boraginaceae**: *Lithospermumzollingeri*	**1**	2	5	10	LBM	in
				*higoniae*	**Boraginaceae**: *Callicarpamollis*	1	1	1	**1**	LBM	in
				*adinae* sp. nov.	**Rubiaceae**: *Adinapilulifera*	1	1	1	**1**	LBM	in
				*issikii*	**Poaceae**: Pleioblastuschinovar.viridis, *P.simonii*	1	1	**1**	2	LBM	in
			*Hispellinus*	*moerens*	**Poaceae**: *Miscanthussinensis*†	1	1	1	**1**	LBM	in
			*Rhadinosa*	*nigrocyanea*	**Poaceae**: *Miscanthussinensis*, *M.condensatus*, *M.tinctorius*, *M.oligostachyus*, Pleioblastuschinovar.viridis	1	**1**	2	5	LBM	in
			*Platypria*	*melli*	**Rhamnaceae**: *Hoveniadulcis*	1	1	1	**1**	BM	in
			*Dicladispa*	*boutani*	**Poaceae**: *Oryzasativa*†	1	1	1	**1**	LBM	in
		Notosacanthini									
			*Notosacantha*	*ihai*	**Proteaceae**: *Heliciacochinchinensis*; **Pentaphylacaceae**: *Adinandraryukyuensis*, Euryajaponicavar.japonica; **Theaceae**: *Schimaliukiuensis*; Staphyleaceae: *Turpiniaternata*; Melastomataceae: *Brediaokinawensis*, *B.yaeyamensis*; **Symplocaceae**: *Symplocosglauca*, *S.sonoharae*; **Loganiaceae**: *Gardnerialiukiuensis*	4	7	8	10	RM	in
				*loochooana*	**Iteaceae**: *Iteaoldhamii*; **Rubiaceae**: *Gardeniajasminoides*	2	2	2	2	RM	in
				*nishiyamai*	**Rubiaceae**: *Coptosapeltadiffusa, Gardeniajasminoides*, *Randiacanthioides*, *Tarennagracilipes*	1	**1**	4	4	RM	in

† host plant species reported by refecences. * LM, linear mine; BM, blotch mine; LBM, linear–blotch mine; RM, radiate mine; PM, petiole/midrib mine; ↓, leaf fall. ** Pupation site: in, internal; ex, external.

### ﻿Cerambycidae Latreille, 1802

**Lamiinae Latreille, 1825**

***Mimectatina* (Matsushita, 1933)**

#### ﻿ Mimectatina meridiana ohirai

Taxon classificationAnimaliaColeoptera﻿Cerambycidae

Breuning & Villiers, 1973

3BCEB7F9-77B7-5ED0-B9A4-56E58F390465

Fig. 1A–F

##### Host plant.

**Figure 1.**
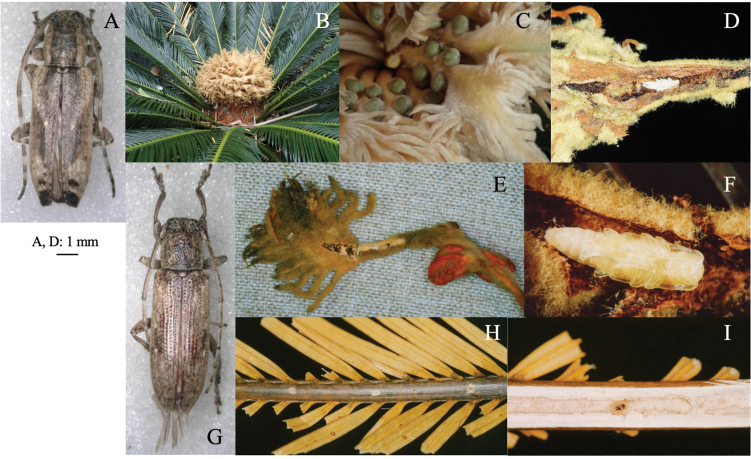
Two cerambycid species associated with *Cycas revouta: Mimectatinameridianaohirai* (**A–F**) and *Sybraflavostriataflavostriata* (**G–I**). **A, G** adult habitus **B** flowering cycad **C** flowering megasporophyll **D** cross section of mined megasporophyll **E** mine of megasporophyll **F** pupa in the mine **H** oviposition scars on leaf stalk **I** larva mining the leaf stalk.

Cycadaceae: *Cycasrevoluta* Thunb. (Fig. 1B–F) (Kato 2001). The wood tissues of *Toddaliaasiatica* (L.) and *Aucubajaponica* Thunb. are used as hosts (Coleopterological Society of Japan 1984).

##### Leaf mine.

Linear or linear-blotch mines in the megasporophyll of female cycad plants (Fig. 1E). The larva sometimes penetrates the testa of the cycad seed. Pupation occurs within the mine on the leaf stalk of the megasporophyll (Fig. 1D, F). Frass is granular and deposited within the mine.

##### Material examined.

• 25 adults, Hedo, Kunigami, Okinawa Is., Okinawa Pref., 1-II-1998 (collected as larva on *Cycasrevoluta*), emerged on 3-III–20-VI-1998; • 7 adults, Hedo, Kunigami, Okinawa Is., Okinawa Pref., 10-III-1997 (as larva on *C.revoluta*), emerged on 25-IV–20-VI-1997; 2 adults, Hedo, Kunigami, Okinawa Is., Okinawa Pref., 19-IV-2000 (as larva on *C.revoluta*), emerged on ?-V-2000; • 8 adults, Angyaba, Kakeroma Is., Setouchi, Kagoshima Pref., 10-V-2001 (as larva on *C.revoluta*), emerged on 12–28-VI-2001.

### ﻿*Sybra* Pascoe, 1865

#### ﻿ Sybra ordinata

Taxon classificationAnimaliaColeoptera﻿Cerambycidae

Bates, 1873

1815AF37-60B1-5689-BB69-0C3626FB9ED5

Fig. 1G–I

##### Host plant.

Cycadaceae: *Cycasrevoluta* (Fig. 1H, I) (Kato 2001). Wood of *Ficussuperba* (Miq.), *Ficuserecta* Thunb., *Pittosporumtobira* (Thunb.), and *Boehmeriabiloba* Wedd. are also used as larval hosts (Coleopterological Society of Japan 1984).

##### Leaf mines.

Linear mine in a leaf stalk. The adult female bites the lower surface of the leaf stalk and inserts eggs into the scar (Fig. 1H). The larva mines the woody leaf stalk linearly, but does not enter leaflets. The fully grown larva pupates in the mine (Fig. 1I).

##### Material examined.

• 5 adults, Haneji, Nago, Okinawa Is., Okinawa Pref., 1-II-1998 (as larva on *Cycasrevoluta*), emerged on ?-V-1998; 2 adults, Hedo, Kunigami, Okinawa Is., Okinawa Pref., 1-II-1998 (as larva on *Cycasrevoluta*), emerged on 20-III-1998.

### ﻿Megalopodidae Latreille, 1802

***Zeugophorinae* Böving & Craighead, 1931**

***Zeugophora* Kunze, 1818**

#### ﻿ Zeugophora annulata

Taxon classificationAnimaliaColeoptera﻿Megalopodidae

(Baly, 1873)

0D5C15D5-09E7-5E75-8312-0BB791EBA2AD

Fig. 2N–Q

##### Host plant.

Celastraceae: *Celastrusorbiculatus* Thunb., *Euonymusalatus* (Thunb.), *E.fortunei* (Turcz.), *E.japonicus* Thunb., *E.macropterus* Rupr., *E.melananthus* (Thunb.), *E.oxyphyllus* Miq., *E.tricarpus* Koidz., *E.sieboldianus* Blume. *Tripterygiumregelii* is also recorded as a host by Takemoto (2019).

##### Leaf mine.

**Figure 2.**
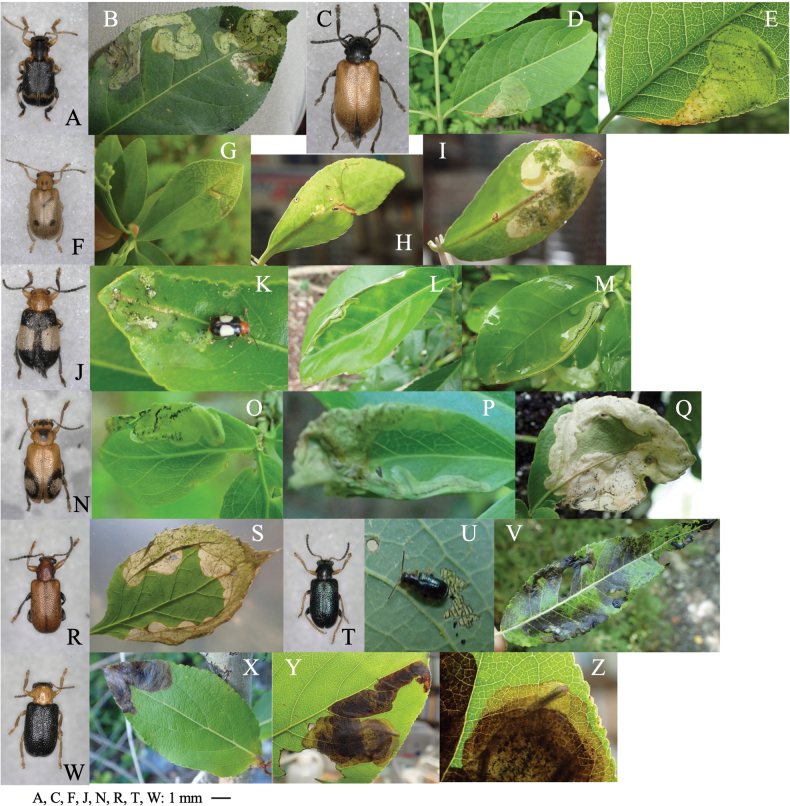
Habitus and feeding scars of adult beetles and leaf mines of eight *Zeugophora* species **A, B***Z.nigricolis***C–E***Z.unifasciata***F–I***Z.chujoi***J–M***Z.flavonotata***N–Q***Z.annulata***R, S***Z.varipes***T–V***Z.hozumii***W–Z***Z.japonica*. Host plants **A, B***Euonymussieboldianus* at Hirayu, Gifu Pref. **C–E***E.sieboldianus* at Sugadaira, Nagano Pref. **F–I***E.fortunei* at Nagao-toge, Shizuoka Pref. **J–L***E.tanakae* at Yutsun, Iriomote Is., Okinawa Pref. **M***E.japonicus* at Hoshidate, Iriomote Is., Okinawa Pref. **N, O***E.alatus* at Kiyosato, Yamanashi Pref. **P***E.melananthus* at Mt. Tara, Nagasaki Pref. **Q***E.fortunei* at Mt. Myôjô, Niigata Pref. **R, S***Symplocossawafutagi* at Iyari, Nagano Pref. **T–V**Salixcardiophyllavar.urbaniana at Kitazawa-toge, Nagano Pref. **W–Z***Populussuaveolens* at Sôunkyô, Hokkaido.

Full-depth linear-blotch mine on newly-opened leaf (Fig. 2O–Q). The mine often covers the whole area of the leaf causing deformation of the leaf, and the larva moves to another unmined leaf. Frass is thread-like and meandering, deposited along the center of the mine. The fully grown larva exits the mined leaf, falls to the ground, and pupates underground.

##### Material examined.

• 4 adults, Iyari, Ômachi, Nagano Pref., 5-V-2016 (as larva on *Euonymusalatus*), emerged on 27-V-2016 (Fig. 2N, O); 5 adults, Saroma Lake, Yûbetsu, Hokkaidô, 5-VI-2016 (as larva on *E.sieboldianus*), emerged on 29–30-VI-2016; 3 adults, Mt. Tara, Isahaya, Nagasaki Pref., 19-IX-2017 (as larva on *E.melananthus*), emerged on 23-V-2017 (Fig. 2P); • 2 adults, Mt. Myôjô, Niigata Pref., Aoi-ku, Shizuoka Pref., 9-V-2018 (as larva on *E.fortunei*), emerged on 10-VI-2018 (Fig. 2Q); • 1 adult, Mt. Teine, Sapporo, Hokkaido, 6-VI-2016 (as larva on *E.tricarpus*), emerged on 25-VI-2016; • 2 adults, Ashiu, Nantan, Kyoto Pref., 5-V-1992 (as larva on *E.oxyphyllus*), emerged on ?-V-1992; • 1 adult, Hidakatsu, Tsushima Is., Nagasaki Pref., 19-IV-2009 (as larva on *E.japonicus*), emerged on 18-V-2009; • 1 adult and many leaf mines, Mt. Byôbu, Kizukuri, Aomori Pref., 14-V-1993 on *E.macropterus*; • 3 adults, Misakubo Dam, Hamamatsu, Shizuoka Pref., 10-V-2000 (as larva on *Celastrusorbiculatus* collected by T. Kato), emerged on 7-VI-2000.

#### ﻿ Zeugophora chujoi

Taxon classificationAnimaliaColeoptera﻿Megalopodidae

Ohno, 1961

19F7DD9E-BC34-5900-9C1C-EF331BE3772B

Fig. 2F–I

##### Host plant.

Celastraceae: *Euonymusfortunei* (Turcz.).

##### Leaf mine.

Full-depth linear-blotch mine on the leaf blade and the midrib of a newly-opened leaf (Fig. 2G–I). The egg is laid in the midrib and the hatched larva enters the midrib and bores into the petiole, leading to leaf abscission from the shoot at the petiole base. Following the leaf fall, the mine diverges from the midrib and expands in the leaf blade, forming a blotch mine. Frass is granular and scattered throughout the mine. The fully grown larva exits the mined leaf, falls to the ground, and pupates underground.

##### Material examined.

• 4 adults, Nagao-tôge, Gotenba, Shizuoka Pref., 15-V-2018 (as larva on *Euonymusfortunei*), emerged on 18-VI-2018 (Fig. 2F–I).

#### ﻿ Zeugophora flavonotata

Taxon classificationAnimaliaColeoptera﻿Megalopodidae

(Chûjô, 1935)

BDE06BAD-F2C6-51C5-8BF1-A386EF1984F9

Fig. 2J–M

##### Host plant.

Celastraceae: *Euonymuscarnosus* Maxim., *E.japonicus* Thunb.

##### Leaf mine.

Full-depth linear mine on the newly-opened leaf (Fig. 2K–M). The egg is laid near the leaf margin and the hatched larva mines along the leaf margin, slowly expanding the mine. Frass is thread-like, deposited somewhat to one side of the center. The fully grown larva exits the mined leaf, falls to the ground, and pupates underground.

##### Material examined.

• 2 adults, Yutsun, Iriomote Is., Yaeyama, Okinawa Pref., 24-III-2022 (as larva on *Euonymuscarnosus*), emerged on 16–18-IV-2022 (Fig. 2J–L); 3 adults, Todomari, Iriomote Is., Yaeyama, Okinawa Pref., 27-III-2018 (as larva on *E.carnosus*), emerged on 16–18-IV-2018; 14 adults, Hoshidate, Iriomote Is., Yaeyama, Okinawa Pref., 6-III-2019 (as larva on *E.japonicus*), emerged on 3–6-IV-2019 (Fig. 2M).

#### ﻿ Zeugophora nigricolis

Taxon classificationAnimaliaColeoptera﻿Megalopodidae

(Jacoby, 1885)

B6C5DBBD-BD75-5272-B54E-55022F53A3E2

Fig. 2C–E

##### Host plant.

Celastraceae: *Euonymussieboldianus* Blume.

##### Leaf mine.

Upper-layer ophiogenous blotch mine on the mature leaf (Fig. 2D, E). Within the blotch, the larval trajectory itself is linear and meandering compactly. The egg is laid along the basal margin of the leaf and the hatched larva mines along the leaf margin, abruptly expanding the mine. Within the blotch, the larval trajectory itself is linear and meandering compactly. Frass is granular and deposited along the meandering trajectory. The fully grown larva exits the mined leaf, falls to the ground, and pupates underground.

##### Material examined.

• 2 adults, Sugadaira, Ueda, Nagano Pref., 18-VI-2017 (as larva on *Euonymussieboldianus*), emerged on 20–28-VII-2017 (Fig. 2C–E).

#### ﻿ Zeugophora unifasciata

Taxon classificationAnimaliaColeoptera﻿Megalopodidae

(Jacoby, 1885)

0A80E343-CF8C-5414-A746-8F66C9AB7B5C

Fig. 2A, B

##### Host plant.

Celastraceae: *Euonymussieboldianus*.

##### Leaf mine.

Upper-layer linear mine on mature leaf (Fig. 2B). The egg is laid in the leaf blade and the hatched larva construct slightly meandering mine. Width of the mine is slightly wider than the width of the larva. Frass is thread-like, deposited along the meandering trajectory. The fully grown larva exits the mined leaf, falls to the ground, and pupates underground.

##### Material examined.

• 5 adults, Hirayu-tôge, Takayama, Gifu Pref., 6-IX-2019 (as larva on *Euonymussieboldianus*), emerged on 6–7-X-2019 (Fig. 2A, B); • 1 adult, Toyohara, Nasu, Tochigi Pref., 2-VII-2022 (as larva on *E.sieboldianus*), emerged on 3-VIII-2022.

#### ﻿ Zeugophora varipes

Taxon classificationAnimaliaColeoptera﻿Megalopodidae

(Jacoby, 1885)

B21F7B41-4516-5A72-8F23-4E163359BA0E

Fig. 2R, S

##### Host plant.

Symplocaceae: *Symplocoscoreana* (H. Lev.), *S.sawafutagi* Nagam.

##### Leaf mine.

Full-depth linear-blotch mine on young leaf (Fig. 2S). The egg is laid in the leaf blade, and the hatched larva constructs linear mine along the leaf margin, then gradually expands the mine. The mine is wider than the width of the larva. Frass is granular, scattered throughout the mine. The fully grown larva exits the mined leaf, falls to the ground, and pupates underground.

##### Material examined.

• 1 adult and 5 leaf mines, Iyari, Ômachi, Nagano Pref., 5-V-2016 on *Symplocossawafutagi* (Fig. 2R, S); 6 adults, Omotsubo, Okayama Pref., 27-V-1993 (as larva on *Symplocoscoreana*), emerged on 20–24-VI-1993.

#### ﻿ Zeugophora hozumii

Taxon classificationAnimaliaColeoptera﻿Megalopodidae

Chûjô, 1953

DBE888AD-23FD-5D62-AF7F-6C5C2F140703

Fig. 2T–V

##### Host plant.

Salicaceae: *Salixcardiophylla* Trautv. et C. A. Mey. Although Takemoto (2019) lists *Salixcaprea* L. as a host plant, this record is not confirmed.

##### Leaf mine.

Black upper-layer linear-blotch mine on mature leaf (Fig. 2V). The egg is laid in the leaf blade, and the hatched larva constructs a linear-blotch mine, the upper surface of which turns black. Frass is granular, scattered throughout the mine. The fully grown larva exits the mined leaf, falls to the ground, pupates underground, and hibernates as a pupa.

##### Material examined.

• 8 adults and many leaf mines, Kitazawa Tôge, Ina, Nagano Pref., 30-VII-2016, feeding lower surface of leaf of *Salixcardiophylla* (Fig. 2T–V); • 1 adult, Azusa-gawa, Matsumoto, Nagano Pref., 24-X-2020 (as larva on *Salixcardiophylla*), emerged on 22-III-2021.

#### ﻿ Zeugophora japonica

Taxon classificationAnimaliaColeoptera﻿Megalopodidae

Chûjô, 1951

9861A8A9-1048-5893-BF21-C99E21F2DF55

Fig. 2W–Z

##### Host plant.

Salicaceae: *Populussuaveolens* Fisch.

##### Leaf mine.

Black upper-layer blotch mine on mature leaf, sometimes gregarious (Fig. 2X–Z). The egg is laid along the leaf margin, and the hatched larva constructs a blotch mine, the upper surface of which turns black. Frass is granular, scattered throughout the mine. The fully grown larva exits the mined leaf, falls to the ground, pupates underground.

##### Material examined.

• 1 adult, Rekifune Nakanokawa, Taiki, Tokachi, Hokkaidô, 23-VI-2017 (as larva on *Populussuaveolens*), emerged on 21-VIII-2017 (Fig. 2W–Z); • 2 adults, Rubeshibe, Kitami, Hokkaidô, 25-VI-2017 (as larva on *P.suaveolens*), emerged on 21-VIII-2017.

#### ﻿ Zeugophora cupka

Taxon classificationAnimaliaColeoptera﻿Megalopodidae

Takemoto, 2019

487307BF-F38B-5D06-B184-3170E1E7811F

##### Host plant.

Salicaceae: *Populussuaveolens* (Takemoto 2019). Leaf mine has not yet been reported.

#### ﻿ Zeugophora gracilis

Taxon classificationAnimaliaColeoptera﻿Megalopodidae

Chûjô, 1958

B9548D24-2A9A-57CC-A40D-D5481FEFE7B0

##### Note.

There is no recent collection of this species, and its host plant and mine are not known (Takemoto 2019).

### ﻿Chrysomelidae Latreille, 1802

**Galerucinae Latreille, 1802**

**Alticini Newman, 1834**

***Phyllotreta* Chevrolat, 1836**

#### ﻿ Phyllotreta ezoensis

Taxon classificationAnimaliaColeopteraChrysomelidae

Kimoto, 1993

57C3B260-E46B-5028-A2D6-9C919A668008

Fig. 3A–I

##### Host plant.

**Figure 3.**
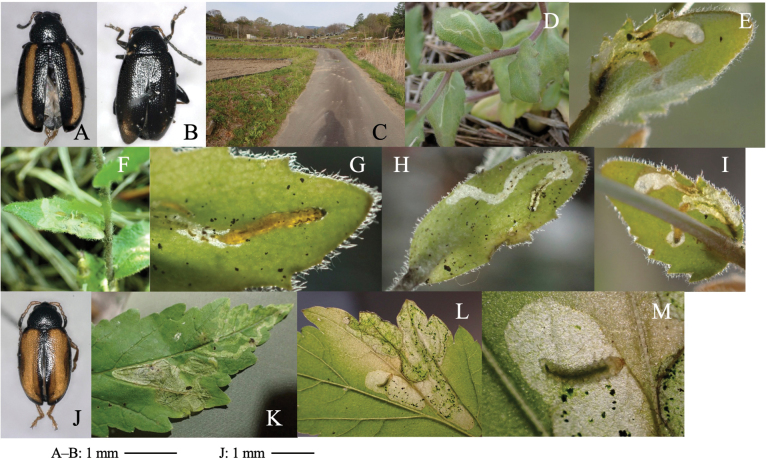
Habitus of adults and leaf mines of two *Phyllotreta* species **A–I***P.ezoensis***J–M***P.shirahatai.* Host plants **A–I***Drabanemorosa* at Kiyosato, Yamanashi Pref. **J–M***Cardamineappendiculata* at Eniwa (**J, K**) and Tobai (**L, M**), Hokkaido.

Brassicaceae: *Drabanemorosa* L. The host plant grows on levee of traditional rice fields and upland fields in Central Honshu (Fig. 3C).

##### Leaf mine.

Full-depth linear mine of leaf blade, midrib, petiole, and shoot (Fig. 3D–I). The egg is laid on the leaf, and the hatched larva mines toward the midrib, and reenters midrib/leaf blade. Larvae repeats mining leaf and mining midrib, petiole, and/or shoot, often by exiting its mine and establishing a new mine. Frass is granular, deposited linearly along midline of the mine. The fully grown larva exits the mined leaf, falls to the ground, and pupates underground. The adult emerges ~ 2 weeks after pupation, and varies in elytral pattern (Fig. 3A, B).

##### Material examined.

• 6 adults, Matsubara-Lake, Koumi, Nagano Pref. 21-V-2021 (as larva on *Drabanemorosa*), emerged on 4-VI-2021 (Fig. 3A–E); • 15 adults, Kiyosato, Hokuto, Yamanashi Pref., 4-V-2022 (as larva on *D.nemorosa*), emerged on 17–20-V-2022 (Fig. 3F–I).

#### ﻿ Phyllotreta shirahatai

Taxon classificationAnimaliaColeopteraChrysomelidae

Madar, 1959

E47EC518-EAA5-5834-B80B-A48FB3EDF243

Fig. 3J–M

##### Host plant.

Brassicaceae: *Cardamineleucantha* (Tausch.).

##### Leaf mine.

Full-depth linear-blotch mine on mature leaf (Fig. 3K–M). Frass is granular, scattered throughout the mine. The fully grown larva exits the mined leaf, falls to the ground, and pupates underground.

##### Material examined.

• 1 adult and several leaf mines on *Cardamineleucantha*, Eniwa Park, Eniwa, Hokkaidô, 21-VII-2020 (Fig. 3J, K); several leaf mines, Tôbai, Nemuro, Hokkaidô, 2-VIII-2023 on *C.leucantha* (Fig. 3L, M).

### ﻿*Longitarsus* Latreille, 1829

#### ﻿ Longitarsus aff. holsaticus

Taxon classificationAnimaliaColeopteraChrysomelidae

(Linnaeus, 1758)

3803BDD8-6D85-5A85-BC88-5C1EBEF24EEB

Fig. 4A–G

##### Note.

Whereas six collected specimens were tentatively identified as this species, more specimens and further taxonomic studies are necessary.

##### Host plant.

Plantaginaceae: *Pennellianthusfrutescens* (Lamb.). Host plants of *Longitarsusholsaticus* are reported to be *Veronica* spp. by Kimoto and Takizawa (1993).

##### Leaf mine.

**Figure 4.**
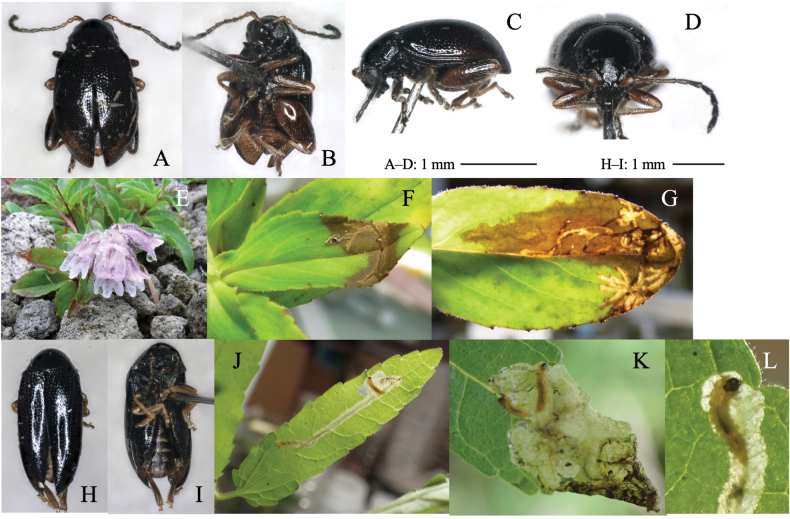
Habitus of adults and leaf mines of two species belonging to two Alticini genera, *Longitausus and Dibolia***A–G***Longitarsusholsaticus***H–L***Diboliajaponica.* Host plants **A–G***Pennellianthusfrutescens* at Mt. Tarumae, Hokkaido **H–L**Stachysasperavar.hispidula at Lake Shoji-ko, Yamanashi Pref.

Full-depth linear or radiate mine in young leaf (Fig. 4F–G). Frass is deposited compactly in a few parts of the mine, and sometimes discharged from the mine through holes perforated along the mine edge. The fully grown larva exits the mined leaf, falls to the ground, and pupates underground.

##### Material examined.

• 6 adults, Mt. Tarumai, Chitose, Hokkaidô, 4-VII-2010 (as larva on *Pennellianthusfrutescens*), emerged on 17-VIII-2010 (Fig. 4A–G).

### ﻿*Dibolia* Latreille, 1829

#### ﻿ Dibolia japonica

Taxon classificationAnimaliaColeopteraChrysomelidae

S.-H. Chen, 1933

B7DDF71D-D6CA-5577-A75E-E8481A7DCDE3

Fig. 4H–L

##### Host plant.

Lamiaceae: *Stachysaspera* Michx.

##### Leaf mine.

Full-depth linear-blotch mine on mature leaf (Fig. 4J–L). Frass is thread-like, deposited along trajectory in the mine. The fully grown larva exits the mined leaf, falls to the ground, pupates underground.

##### Material examined.

• 1 adult, Shôji-ko Lake, Fuji-kawaguchiko, Yamanashi Pref., 4-VII-2010 (as larva on *Stachysaspera*), emerged on 3-VIII-2010 (Fig. 4H–L).

### ﻿*Mantura* Stephens, 1831

#### ﻿ Mantura clavareaui

Taxon classificationAnimaliaColeopteraChrysomelidae

Heikertinger, 1912

C74E1ADF-C922-5B71-9A3D-0EC1B2443B1F

Fig. 5A–I

##### Host plant.

Polygonaceae: *Rumexjaponicus* Houtt., *Polygonumaviculare* L. *Rumexacetosa* L. is also recorded as a host plant (Kimoto and Takizawa 1993).

##### Leaf mine.

**Figure 5.**
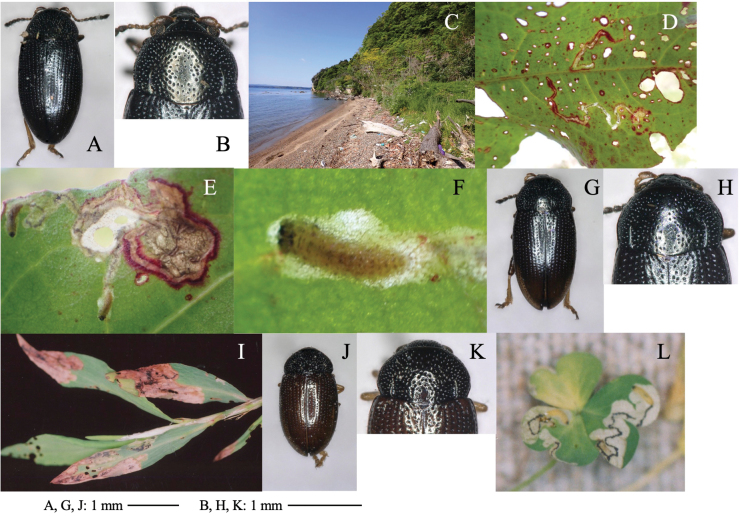
Habitus of adults, habitat, and leaf mines of two *Mantura* species **A–I***M.clavareaui***J–L***M.fulvipes*. Host plants **A–F***Rumexjaponicus* at Notojima Island, Ishikawa Pref. **G–I***Polygonumaviculare* at Mihogaseki, Shimane Pref. **J–L***Oxaliscorniculata* at Tamanoura, Fukue Is., Nagasaki Pref.

Full-depth linear mine occurs on mature leaf (Fig. 5D–F, I). The larva alternates mining the leaf blade and mining the midrib, the petiole, and the shoot, and often relocates its mine. Frass is granular, deposited linearly along middle line of the mine. The fully grown larva exits the mined leaf, falls to the ground, and pupates underground.

##### Material examined.

• 3 adults, Kôda, Notojima Is., Nanao, Ishikawa Pref. 2-V-2023 (as larva on *Rumexjaponicus*), emerged on 22-V-2023 (Fig. 5A–F); • 21 adults, Kamematsu, Shivani, Kumamoto Pref., 28-IV-2017 (as larva on *R.japonicus*), emerged on 13–14-V-2017; • 14 adults, Mihonoseki, Matsue, Shimane Pref., 15-V-2006 (as larva on *Polygonumaviculare*), emerged on 31-V–3-VI-2006 (Fig. 5G–I).

#### ﻿ Mantura fulvipes

Taxon classificationAnimaliaColeopteraChrysomelidae

Jacoby, 1885

66926E35-CC8A-5B9C-A0A5-C113DFDD4DCA

Fig. 5J–L

##### Host plant.

Oxalidaceae: *Oxaliscorniculata* L.

##### Leaf mine.

Full-depth linear mine on mature leaf (Fig. 5L). The egg is laid along the leaf margin, and hatched larva mines along the leaf margin, gradually expanding the mine. Width of the mine is wider than that of larva. Frass is thread-like, deposited along middle line of the mine. The fully grown larva exits the mined leaf, falls to the ground, pupates underground.

##### Material examined.

• 19 adults, Tamanoura, Fukue Is. Goto, Nagasaki Pref. 9-X-1998 (as larva on *Oxaliscorniculata*), emerged on 22–26-X-1998 (Fig. 5J–L).

#### ﻿ Mantura japonica

Taxon classificationAnimaliaColeopteraChrysomelidae

Jacoby, 1885

649F7497-9C60-5B89-8415-7320420F487C

##### Note.

Host plant has not been reported (Hayashi et al. 1984; Kimoto and Takizawa 1993).

### ﻿*Hippuriphila* Foudras, 1859

#### ﻿ Hippuriphila babai

Taxon classificationAnimaliaColeopteraChrysomelidae

(Chûjô, 1959)

2A706F2C-7988-5682-A69F-00361CF737C2

Fig. 6A–F

##### Host plant.

Equisetaceae: *Equisetumarvense* L., *E.fluviatile* L.

##### Leaf mine.

**Figure 6.**
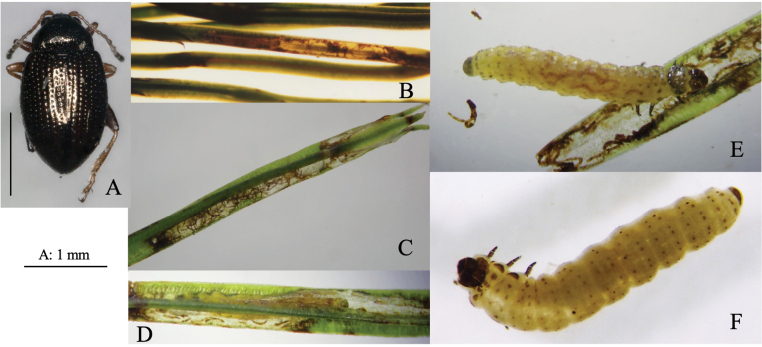
Habitus of adults, leaf mine, and larva of *Hippuriphilababai* on *Equisetumarvense***A** habitus of adults **B, C** leaf-mine at Mt. Teine **D–F** larva at Yubari, Hokkaido.

Full-depth or internal linear mine on leaf-like branch and the green stem (Fig. 6B–E). The larva is cylindrical (Fig. 6F) and sometimes exits its mine and moves to the other shoot. Frass is thread-like, deposited in the mine.

##### Material examined.

• 2 adults, Mt. Teine, Sapporo, Hokkaidô, 9-X-1998 (as larva on *Equisetumarvense*), emerged on 25-VII–3-VIII-1998 (Fig. 6A–C); • 2 adults, Rebun Is., Hokkaidô, 9-VII-1995 (as larva on *E.arvense*), emerged on 25–27-VII-1995; • 5 adults, Bibi, Chitose, Hokkaidô, 30-VI-2023 (as larva on *E.fluviatile*), emerged on 17–20-VII-2023.

### ﻿*Psylliodes* Latreille, 1829

#### ﻿ Psylliodes punctifrons

Taxon classificationAnimaliaColeopteraChrysomelidae

Baly, 1874

1871083B-ACA4-5D32-9BB6-12C3A7B3024A

Fig. 7A–P

##### Host plant.

Brassicaceae: *Brassicajuncea* (L.), RaphanussativusL.var.raphanistroides (Makino), *Rorippapalustris* (L.), *Eutremajaponicum* (Miq.). *Cardamineanemonoides*, *Brassicachinensis* and *B.napus* are also recorded as host plants (Takizawa 2005).

##### Leaf mine.

**Figure 7.**
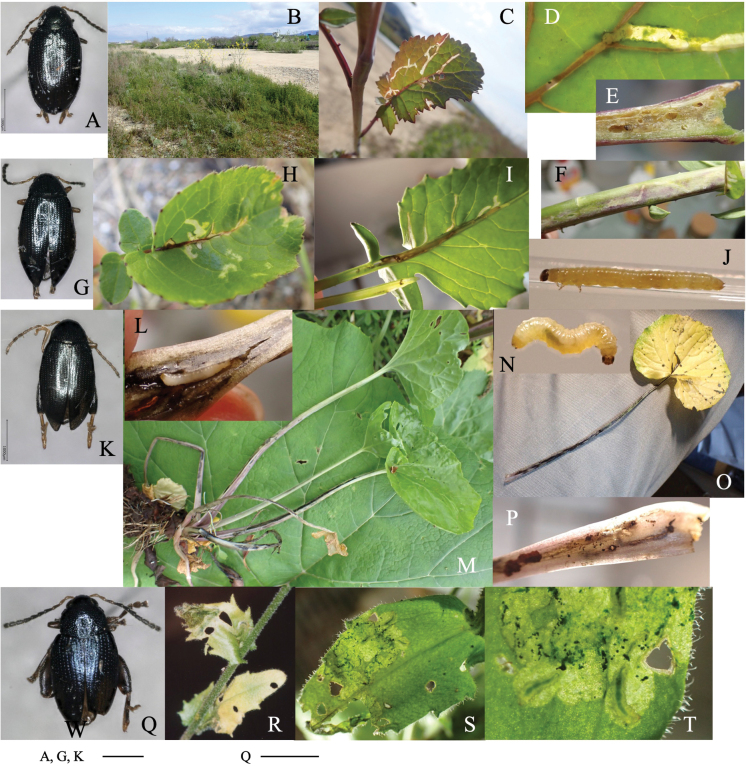
Habitus of adults and larvae, habitat, and leaf mines of three *Psylliodes* species **A–J***P.punctifrons***K–P***P.sasakii***Q–T***P.subrugosa.* Host plants **A–F***Brassicajuncea* at Kizu-gawa, Kyôto **G–I**Raphanussativusvar.hortensis at Notojima Is., Ishikawa Pref. **K–M***Eutremajaponicum* at Futamata, Oshamanbe, Hokkaido **N–P***Eutremajaponicum* at Nyûkawa, Takayama, Gifu Pref. **Q–T***Arabishirsuta* at Shimo-suwa, Nagano Pref.

Full-depth linear mine occurs on leaf blade, midrib, petiole, and shoot (Fig. 7C–E, H, I, L–P). The egg is laid on the leaf, and the hatched larva mines toward midrib, and reenters leaf blade. Larvae alternates mining the leaf and mining the midrib/petiole/shoot, and often relocates its mine. Frass is deposited compactly in a few parts of the mine, and sometimes discharged from the mine through the perforated holes. The fully grown larva exits the mined leaf, falls to the ground, pupates underground. Adult emerges ~ 1 month after pupation. On *Eutremajaponicum* with long petiole, linear mine of petiole and midrib of mature leaf (Fig. 7L–P). The mined petiole turns blackish. Frass is deposited compactly in a few parts of the mine, and sometimes discharged from the mine through perforated holes.

##### Material examined.

• 23 adults, Kusauchi, Kizu-gawa, Kyôtanabe, Kyoto Pref. 11-III-2020 (as larva on *Brassicajuncea*), emerged on 1–15-V-2020 (Fig. 7A–E); • 1 adult, Kôda, Notojima Is., Nanao, Ishikawa Pref., 2-V-2023 (as larva on Raphanussativusvar.raphanistroides), emerged on 4-VI-2023 (Fig. 7G–J); • 2 adults, Watarase, Fujioka, Tochigi Pref., 25-V-2020 (as larva on *Rorippapalustris*), emerged on 8–14-VI-2020; • 19 adults, Futamata, Oshamanbe, Hokkaidô, 20-V-2023 (as larva on *Eutremajaponicum*), emerged on 14–25-VI-2023 (Fig. 7K–M); • 2 adults, Nyû-kawa, Takayama, Gifu Pref., 1-V-2023 (as larva on *Eutremajaponicum*), emerged on 29-V–5-VI-2023 (Fig. 7N–P).

#### ﻿ Psylliodes aff. subrugosa

Taxon classificationAnimaliaColeopteraChrysomelidae

Jacoby, 1885

979B4F41-63E7-5873-A503-C4D9A3674176

Fig. 7Q–T

##### Note.

Two female specimens were reared and tentatively identified as related to this species, but there is a possibility that this is an undescribed species.

##### Host plant.

Brassicaceae: *Arabishirsuta* (L.). Crucifers are known as host plants (Kimoto and Takizawa 1993), while its biology has not been reported.

##### Leaf mine.

Linear mine in the midrib and the petiole of mature leaf (Fig. 7R–T). The egg is laid on the petiole, and the hatched larva mines the petiole, and sometimes enter the midrib. The mined petiole becomes blackish. Frass is deposited compactly in a few parts of the mine, and sometimes discharged from the mine through perforated holes. The fully grown larva (Fig. 7N) exits the mined leaf, falls to the ground, and pupates underground. The adult emerges ~ 1 month after pupation.

##### Material examined.

• 2 adults, Higashimata, Shimosuwa, Suwa-gun, Nagano Pref., 28-VI-2020 (as larva on *Arabishirsuta*), emerged on 9-VIII-2020 (Fig. 7Q, S, T): several leaf mines on *Arabishirsuta*, Sanjiro, Matsumoto, Nagano Pref., 28-VII-1995 (Fig. 7R).

### ﻿*Halticorcus* Lea, 1917

#### ﻿ Halticorcus kasuga

Taxon classificationAnimaliaColeopteraChrysomelidae

(Nakane, 1963)

C3DA3799-AADD-5FA7-9FA2-27B85AC4132A

Fig. 8A–H

##### Host plant.

Polypodiaceae: *Lepisorusmiyoshianus* (Makino)., *L.onoei* (Franch. et Sav.), *L.thunbergianus* (Kaulf.), *Pyrrosialinearifotia* (Hook.) and *Lemmaphyllummicrophyllum* C. Presl. are reported as adult’s host plants by Suzuki et al. (2008).

##### Leaf mine.

**Figure 8.**
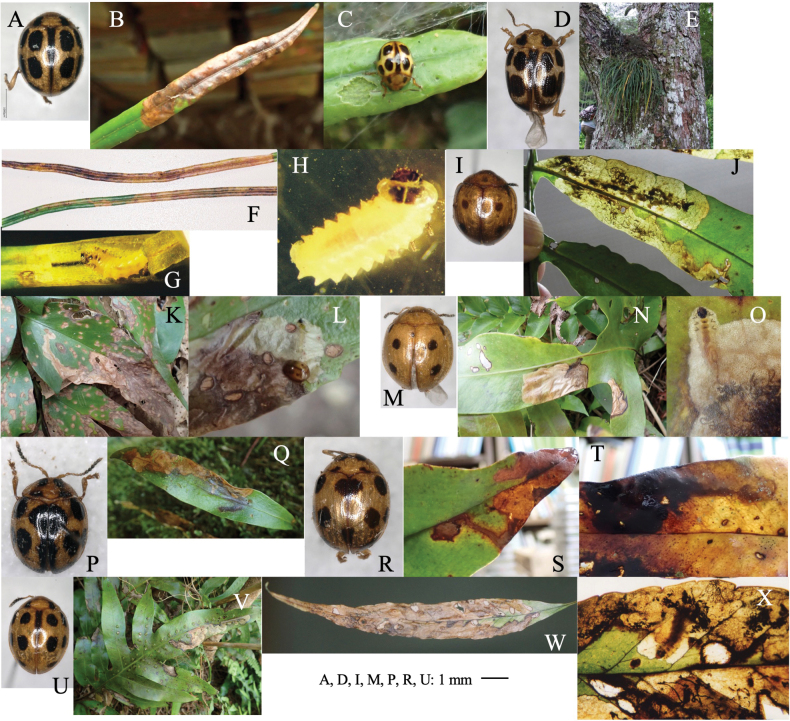
Habitus of adults and leaf mines of four *Halticorcus* species **A–H***H.kasuga***I–L***H.sauteri***M–T***H.hiranoi***U–X***H.duodecimmaculata.* Host plants **A, B***Lepisorusthunbergianus* at Mt. Shizuhata, Shizuoka Pref. **C***Lepisorusonoei* at Shôjiko Lake, Yamanashi Pref. **D–H***L.miyoshianus* at Kiso-fukushima, Nagano Pref. **I–J***Leptochilusneopothifolius* at Kanyû, Kakeroma Is., Kagoshima Pref. **K–L***Cyrtomiumfalcatum* at Imasato, Amami-ôshima Is., Kagoshima Pref. **M–O***Phymatosorusscolopendria* at Tonaki Is., Okinawa Pref. **P, Q***Loxogrammesalicifolia* at Inohae, Kitago, Miyazaki Pref. **R–T***Pyrrosialingua* at Mt. Yuwan, Amami-ôshima Is., Kagoshima Pref. **U, V***Phymatosorusscolopendria* at Iriomote Is., Okinawa Pref. **W, X***Crypsinusyakushimensis* at Iriomote Is., Okinawa Pref.

Upper-layer linear-blotch mine on mature leaf (Fig. 8B, F, G). The fully grown larva (Fig. 8H) exits the mined leaf, falls to the ground, and pupates underground.

##### Material examined.

• 3 adults, Mt. Shizuhata, Aoi-ku, Shizuoka Pref., 27-VII-2017 (as larva on *Lepisorusthunbergianus*), emerged on 12–18-IX-2017 (Fig. 8A, B); • 1 adult on *L.onoei*, Shôji-ko Lake, Fuji-kawaguchi-ko, Yamanashi Pref., 8-IX-2019 (Fig. 7C); • 2 adults, Shimo-shimizu, Kiso-fukushima, Nagano Pref., 7-VIII-2011 (as larva on *L.miyoshianus*), emerged on 4-IX-2011 (Fig. 7D–H).

#### ﻿ Halticorcus sauteri

Taxon classificationAnimaliaColeopteraChrysomelidae

(S.-H. Chen, 1934)

FF28D892-41BB-52E4-8F74-5A0090CD7F51

Fig. 8I–O

##### Host plant.

Dryopteridaceae: *Cyrtomiumfalcatum* (L. f.); Oleandraceae: *Nephrolepiscordifolia* (L.); Polypodiaceae: *Colysiselliptica* (Thunb.), *Loxogrammesalicifolia* (Makino), *Leptochilusneopothifolius* Nakaike, *Lemmaphyllummicrophyllum* Presl., *Microsorumbuergerianum* (Miq.), *Phymatosorusscolopendria* (Burm. f.). *Thelypterisacuminata* (Houtt.) is also reported as adult’s host plants by Suzuki et al. (2008).

##### Leaf mine.

Upper-layer linear-blotch mine on mature leaf (Fig. 8J–L, N, O). Frass is thread-like, thin, and often intermittent, and deposited along the meandering larval trajectory. The fully grown larva exits the mined leaf, falls to the ground, and pupates underground.

##### Material examined.

• 1 adult and several leaf mines, Imasato, Uken, Amami-ôshima Is. Kagoshima Pref., 4-IX-2011 on *Cyrtomiumfalcatum* (Fig. 8K, L); • 2 adults, Angyaba, Tatsugô, Amami-ôshima Is. Kagoshima Pref., 8-V-1997 (as larva on *Cyrtomiumfalcatum*), emerged on 22-VI-1997; • 22 adults, Kinsakubaru, Naze, Amami-ôshima Is. Kagoshima Pref., 3-VI-1996 (as larva on *Nephrolepiscordifolia*), emerged on 2-VII-1996; • 1 adult, Naon, Yamato, Amami-ôshima Is. Kagoshima Pref., 11-V-2002 (as larva on *Colysiselliptica*), emerged on 11-VI-2002; • 10 adults, Segiri, Nagata, Yaku Is., Kumage-gun, Kagoshima Pref., 27-V-1990 (as larva on *Leptochilusneopothifolius*), emerged on ?-VII-1990; • 3 adults, Higashinamaka, Amami-ôshima Is. Kagoshima Pref., 20-V-2015 (as larva on *Leptochilusneopothifolius*), emerged on 17–18-VI-2015 (Fig. 8I–J); • 2 adults, Mt. Yuwan, Uken, Amami-ôshima Is., Kagoshima Pref., 30-VI-1992 (as larva on *Microsorumbuergerianum*), emerged on 25-VII-1992; • 1 adult and several leaf mines, Tonaki Is., Shimajiri-gun, Okinawa Pref., 18-III-2020 (as larva on on *Phymatosorusscolopendria*), emerged on 20-V-2020 (Fig. 8M–O).

#### ﻿ Halticorcus hiranoi

Taxon classificationAnimaliaColeopteraChrysomelidae

(Takizawa, 1982)

2C950025-D8EA-50DC-BE00-2B8553F881BB

Fig. 8P–T

##### Host plant.

Aspleniaceae: *Aspleniumantiquum* Makino; Polypodiaceae: *Pyrrosialingua*, *Lemmaphyllummicrophyllum*, *Lepisorusthunbergianus*, *Leptochilusneopothifolius*, *Loxogrammesalicifolia*; Vittariaceae: *Vittariaflexuosa* Fee.

##### Leaf mine.

Upper-layer linear-blotch mine on mature leaf (Fig. 8Q, S, T). Frass is thread-like, thin, and often intermittent, and deposited along meandering larval trajectory. The fully grown larva exits the mined leaf, falls to the ground, and pupates underground.

##### Material examined.

• 1 adult and several leaf mines, Inohae, Nichinan, Miyazaki Pref., 20-V-2015 on *Loxogrammesalicifolia* (Fig. 8P, Q); • 3 adults, Mt. Yuwan, Uken, Amami-ôshima Is., Kagoshima Pref., 8-III-2000 (as larva on *Pyrrosialingua*), emerged on 14–18-V-2000 (Fig. 8R–T); • 1 adults, Mt. Yuwan, Uken, Amami-ôshima Is., Kagoshima Pref., 16-II-1999 (as larva on *Lepisorusthunbergianus*), emerged on 22-IV-1999; • 4 adult, Mt. Yuwan, Uken, Amami-ôshima Is., Kagoshima Pref., 7-III-2004 (as larva on *Aspleniumantiquum*), emerged on 25-V–5-VI-2004; • 2 adults, Mt. Katsuu-dake, Nago, Okinawa Pref., 23-XII-1989 (as larva on *Lemmaphyllummicrophyllum*), emerged on ?-III-1990; • 1 adult, Inokawa-dake, Tokunoshima Is., Kagoshima Pref., 12-III-2001 (as larva on *Vittariaflexuosa*), emerged on 25-VI-2001; 8 adults, Kuroshima Is., Mishima-mura, Kagoshima Pref., 11-III-1995 (as larva on *Vittariaflexuosa*), emerged on 21–25-V-1995.

#### ﻿ Halticorcus duodecimmaculata

Taxon classificationAnimaliaColeopteraChrysomelidae

(S.-H. Chen, 1934)

3CF83FF4-F430-5FB9-BE2F-36897A39878B

Fig. 8U–X

##### Host plant.

Polypodiaceae: *Phymatosorusscolopendria*, *Selligueayakushimensis* (Makino).

##### Leaf mine.

Full-depth linear-blotch mine on mature leaf (Fig. 8V–X). Frass is thread-like, thin, and often intermittent, and deposited along meandering larval trajectory. The fully grown larva exits the mined leaf, falls to the ground, and pupates underground.

##### Material examined.

• 5 adults, Ômija, Iriomote Is., Yaeyama, Okinawa Pref., 27-III-2018 (as larva on *Phymatosorusscolopendria*), emerged on 25-V–7-VI-2018 (Fig. 8U, V); • 9 adults, Urauchi, Iriomote Is., Yaeyama, Okinawa Pref., 11-V-1999 (as larva on *Selligueayakushimensis* collected by Shirô Kobayashi), emerged on ?-VI-1999 (Fig. 8W, X).

### ﻿*Argopistes* Motschulsky, 1860

#### ﻿ Argopistes coccinelliformis

Taxon classificationAnimaliaColeopteraChrysomelidae

Csiki, 1940

B1A3FFE3-60C2-5789-816F-FB4478FC02C1

Fig. 9A, B

##### Host plant.

Oleaceae: *Ligustrummicranthum* Zucc., *L.ovalifolium* Hassk. *Ligustrumjaponicum* Thunb., *Osmanthusheterophyllus* (G. Don), *O.insularis* Koidz., O.×fortunei Carr. are also recorded as adult hosts (Kimoto and Takizawa 1993). In Ogasawara Islands, this is the only leaf-mining chrysomelid species.

##### Leaf mine.

**Figure 9.**
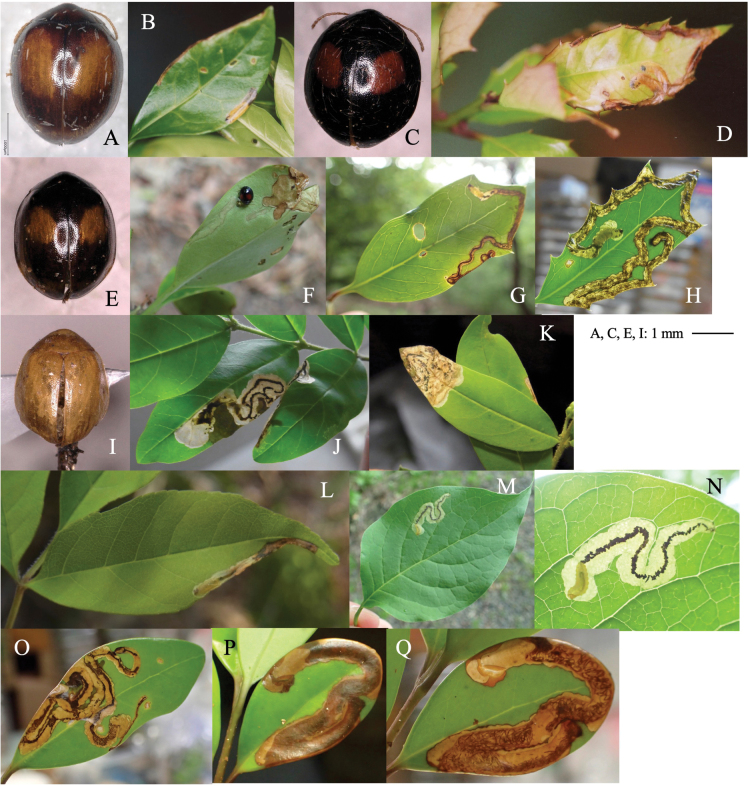
Habitus of adults and leaf mines of four *Argopistes* species **A, B***A.coccinelliformis***C–H***A.biplagiata***I–N***A.tsekooni***O–Q***A.ryukyuensis.* Host plants **A, B***Ligustrumovalifolium* at Shirahama, Wakayama Pref. **C, D***Osmanthus×fortunei* at Mizorogaike Lake, Kyôto Pref. **E–H***Osmanthusheterophyllus* at Iwakura, Kyôto Pref. **I–K***Ligustrumobtusifolium* at Mt Nabejiri, Shiga Pref. **L***Fraxinussieboldiana* at Uri-tôge, Hamamatsu, Shizuoka Pref. **M, N***Syringareticulata* at Tôro Lake, Kushiro, Hokkaido **O–Q***Ligustrumjaponicum* at Abu, Okinawa Is., Okinawa Pref.

Full-depth linear mine on young leaf (Fig. 9B). The egg is laid on leaf margin, and the hatched larva mines adjoining along the leaf margin or its mine. Frass is thread-like, deposited along the middle line of the mine. The fully grown larva exits the mined leaf, falls to the ground, and pupates underground.

##### Material examined.

• 3 adults, Rinkai, Shirahama, Nishimuro-gun, Wakayama Pref., 13-V-1998 (as larva on *Ligustrumovalifolium*), emerged on 2-VI-1998 (Fig. 9A, B); • 1 adult, Mukô-jima Is. Ogasawara, Tokyo Pref. on *Ligustrummicranthum*.

#### ﻿ Argopistes biplagiata

Taxon classificationAnimaliaColeopteraChrysomelidae

Motschulsky, 1860

87504AD5-384F-5E30-B65F-0D33A3F4A28E

Fig. 9C–H

##### Host plant.

Oleaceae: *Ligustrumjaponicum*, *Osmanthusheterophyllus*, *O.insularis*, O.×fortunei.

##### Leaf mine.

Full-depth linear mine on young leaf (Fig. 9D, G, H). The egg is laid on the leaf margin, and the hatched larva mines adjoining along the leaf margin or its mine. Frass is thread-like, deposited along the middle line of the mine in young instars, but in old instars along meandering larval trajectory, scattered throughout the mine. The fully grown larva exits the mined leaf, falls to the ground, and pupates underground.

##### Material examined.

• 15 adults and many leaf mines, Mizorogaike Lake, Sakyo, Kyoto Pref., 13-V-1998 on Osmanthus×fortunei (Fig. 9C, D); • 7 adults, Iwakura, Sakyo, Kyoto Pref., 13-V-2016 (as larva on *Osmanthusheterophyllus*), emerged on 4–8-VI-2016 (Fig. 9E–H).

#### ﻿ Argopistes tsekooni

Taxon classificationAnimaliaColeopteraChrysomelidae

Chen, 1934

CF44E66C-F77E-5DDE-8B10-9B9166E8AB2A

Fig. 9I–N

##### Host plant.

Oleaceae: *Ligustrumobtusifolium* Sieb. et Zucc., *Fraxinussieboldiana* Blume, *Syringareticulata* (Blume).

##### Leaf mine.

Full-depth wide linear mine on mature leaf in young instars and full-depth ophiogenous blotch mine in old instars (Fig. 9J–N). The mine is wider than the larval width, and frass is thick thread-like, deposited linearly along the middle line of the mine. The fully grown larva exits the mined leaf, falls to the ground, and pupates underground.

##### Material examined.

• 3 adults, Mt. Nabejiri, Taga, Shiga Pref., 23-V-2015 (as larva on *Ligustrumobtusifolium*), emerged on ?-VII-2015 (Fig. 9I–K); • 7 adults, Iwakura, Sakyo, Kyoto Pref., 22-IV-2013 (as larva on *Fraxinussieboldiana*), emerged on 3-VI-2013 (Fig. 9L); • 7 adults, Tôro Lake, Shibecha, Kawakami-gun, Kushiro, Hokkaido, 25-VI-2017 (as larva on *Syringareticulata*), emerged on 8-VIII-2017 (Fig. 9M, N).

#### ﻿ Argopistes ryukyuensis

Taxon classificationAnimaliaColeopteraChrysomelidae

Shigetoh & Suenaga, 2022

C88F0506-6577-5F36-952D-6743E62AD719

Fig. 9O–Q

##### Host plant.

Oleaceae: *Ligustrumjaponicum*.

##### Leaf mine.

Full-depth wide linear mine on mature leaf in young instars and full-depth ophiogenous blotch mine in old instars (Fig. 9O–Q). Frass is thread-like, deposited along the middle line of the mine in young instars, but in old instars along meandering larval trajectory, scattered throughout the mine. The fully grown larva exits the mined leaf, falls to the ground, and pupates underground.

##### Material examined.

Several leaf mines, Abu, Nago, Okinawa Is., Okinawa, Pref., 18-V-2017 (Fig. 9O–Q).

#### ﻿ Argopistes unicolor

Taxon classificationAnimaliaColeopteraChrysomelidae

Jacoby, 1885

4F5A951A-720D-5ECF-A2A7-99E900CBA7D9

##### Host plant.

Oleaceae: *Osmanthusheterophyllus* (Hayashi et al. 1984; Kimoto and Takizawa 1993).

##### Leaf mine.

Unknown.

### ﻿*Argopus* Fischer von Waldheim, 1824

#### ﻿ Argopus balyi

Taxon classificationAnimaliaColeopteraChrysomelidae

Harold, 1878

C4A7B25B-00AC-56B1-9C75-F9056A205276

Fig. 10A–D

##### Host plant.

Ranunculaceae: *Clematisstans* Sieb. et Zucc. *C.apiifolia* DC. and *Clematisterniflora* DC. are recorded as adult hosts (Hayashi et al. 1984; Kimoto and Takizawa 1993).

##### Leaf mine.

**Figure 10.**
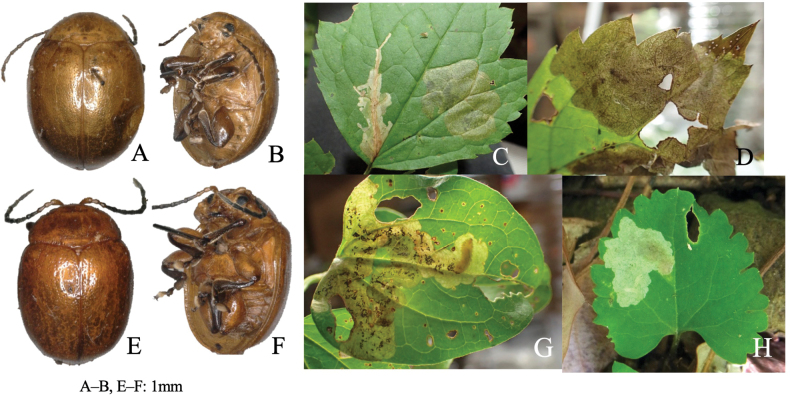
Habitus of adults and leaf mines of four *Argopus* species **A–D***A.balyi***E–H***A.clypeatus.* Host plants **A–D***Clematisstans* at Fukuji-onsen, Gifu Pref. **E–H***Clematisterniflora* (**E–G** at Tsushima Nagasaki Pref., **H** at Uri-tôge, Shizuoka Pref.).

Upper-layer radiate mine along primary leaf vein in young instars (Fig. 10C) and full-depth blotch mine in old instars (Fig. 10D) occur on the mature leaves. Frass is thin threadlike, deposited along meandering larval trajectory in the mine. The fully grown larva exits the mine, falls to the ground, and pupates underground.

##### Material examined.

• 1 adult, Fukuji-onsen, Takayama, Gifu Pref., 3-X-2018 (as larva on *Clematisstans*), emerged on 9-V-2018 (Fig. 10A–D); • 1 adult, Iwakura, Sakyo, Kyoto Pref., 12-X-2009 (as larva on *C.stans*), emerged on 10-V-2009.

#### ﻿ Argopus clarki

Taxon classificationAnimaliaColeopteraChrysomelidae

Jacoby, 1885

9C7557E6-F2C9-5208-93F6-D576CAF736CA

##### Host plant.

Ranunculaceae: *Clematisterniflora* is recorded as larval and adult hosts (Kimoto and Takizawa 1993).

#### ﻿ Argopus clypeatus

Taxon classificationAnimaliaColeopteraChrysomelidae

Baly, 1874

2213D4CC-F511-5003-B3B1-BF5BD107650F

Fig. 10E–H

##### Host plant.

Ranunculaceae: *Clematisterniflora*, *C.apiifolia* (Hayashi et al. 1984; Kimoto and Takizawa 1993).

##### Leaf mine.

Whitish full-depth blotch mine on mature leaf (Fig. 10G, H). Frass is granular, deposited along the larval trajectory in the mine. The fully grown larva exits the mined leaf, falls to the ground, and pupates underground.

##### Material examined.

• 3 adults, Mine, Kami-tsusima Is., Nagasaki Pref., 19-VI-2016 (as larva on *Clematisterniflora*), emerged on 18–29-VIII-2016 (Fig. 10E–G); • 6 adults, Uri-tôge, Mikkabi, Hamamatsu, Shizuoka Pref., 22-IV-2013 (as larva on *C.terniflora*), emerged on 28-V–7-VI-2013 (Fig. 10H); • 5 adults, Nekata, Hamakita, Shizuoka Pref.

#### ﻿ Argopus punctipennis

Taxon classificationAnimaliaColeopteraChrysomelidae

(Motschulsky, 1866)

60A8DB87-45D9-5D18-BB59-CC4E033F9804

Fig. 11A–S

##### Note.

**Figure 11.**
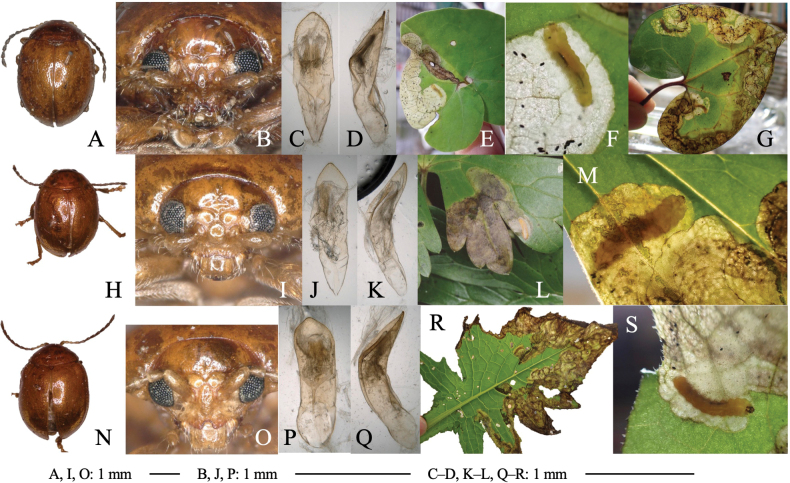
Adult morphology and leaf mines of *Argopuspunctipennis***A–H** on *Asarum***I–N** on *Aconitum***O–S** on *Cirsium*. Host plants **A–F***Asarumheterotropoides* at Samani, Hidaka, Hokkaido **G–H***Asarummegacalyx* at Mt. Haguro, Tsuruoka, Yamagata Pref. **I–M**Aconitumjaponicumsubsp.subcuneatum at Shiriya, Aomori Pref. **N***Aconitumsachalinense* at Hinoemata, Fukushima Pref. **O–R***Cirsiumkamtschaticum* at Aibetsu, Kamikawa, Hokkaido, **S***Cirsiumjaponicum* at Mt. Mikusa, Hyogo Pref. **A, I, O** Habitus **B, J, P** frontal head **C, D, K, L, Q, R** male genitalia in ventral and lateral views **E–G, L, M, R, S** leaf mines.

Leaf-mining larvae of this species have been found from 26 plant species belonging to three taxonomically isolated plant families: Aristolochiaceae (Fig. 11A–G), Ranunculaceae (Fig. 11H–M) and Asteraceae (Fig. 11N–T). The morphology of the adult beetle (Fig. 11A, B, G–H, N–P) and its male genitalia (Fig. 11C, D, J, K, P, Q) suggest that all these specimens belong to this species.

##### Host plant.

Aristolochiaceae: *Asarumasperum* F. Maek., *A.blumei* Duch., *A.caulescens* Maxim., *A.curvistigma* F. Maekawa, *A.heterotropoides* Miq., *A.megacalyx* (F. Maek.), *A.nipponicum* F. Maek., *A.sieboldii* Miq., *A.tohokuense* Yamaji et Ter. Nakam; Ranunculaceae: *Aconitumgigas* H. Lév. et Vaniot, *A.japonicum* Thunb., *A.okuyamae* Nakai, *A.pterocaule* Koidz., *A.sachalinense* F. Schmidt; Asteraceae: *Cirsiumaustrohidakaense* Kadota, *C.iito-kojianum* Kadota, *C.japonicum* Fisch. ex DC., *C.kamtschaticum* Ledeb, *C.kiotoense* (Kitam.), *C.makinoi* Kadota, *C.microspicatum* Nakai, *C.oligophyllum* (Franch. et Sav.), *C.otayae* Kitam., *C.taishakuense* Kadota, *C.ugoense* Nakai, *C.yoshinoi* Nakai.

##### Leaf mine.

Upper-layer linear-blotch mine in young instars, and full-depth blotch mine in old instars occur on mature leaves (Fig. 11E–G, L, M, R, S). Frass is thin thread-like, deposited along meandering larval trajectory in the mine. The fully grown larva exits the mined leaf, falls to the ground, and pupates underground. The mining pattern is largely similar among leaves of the different plant species/families.

##### Material examined.

Aristolochiaceae: • 1 adult, Samani, Hidaka, Hokkaidô, 31-V-2020 on *Asarumheterotropoides* (Fig. 11A, B); • 4 adults, Samani, Hidaka, Hokkaidô, 31-V-2020 (as larva on *Asarumheterotropoides*), emerged on 13–14-VII-2020 (Fig. 11C–E); • 1 adult, Hachimori, Happô, Yamamoto-gun, Akita Pref., 15-VI-2015 (as larva on *Asarumsieboldii*), emerged on ?-VII-2015; • 4 adults, Mt. Haguro, Tsuruoka, Yamagata Pref., 9-VII-2018 (as larva on *Asarummegacalyx*), emerged on 19-VIII–4-IX-2018 (Fig. 11G, H); • 6 adults, Mt. Yakeishi, Ôshû, Iwate Pref., 15-VII-2019 (as larva on *Asarumtohokuense*), emerged on 30-VIII-2018; • 1 adult, Mt. Kiyosumi, Kamogawa, Chiba Pref., 14-V-2008 (as larva on *Asarumnipponicum*), emerged on 20-VII-2008; • 1 adult, Warabino, Aoi-ku, Shizuoka, Shizuoka Pref., 11-V-2004 (as larva on *Asarumcurvistigma*), emerged on 21-VII-2004; • 1 adult, Mt. Gassan, Tsuruoka, Yamagata Pref., 12-VI-2019 (as larva on *Asarumsieboldii*), emerged on 16-V-2019; • 1 adult, Inogashira, Fujinomiya, Shizuoka Pref., 26-V-2002 (as larva on *Asarumcaulescens*), emerged on 4-VII-2002; • 1 adult, Iwakura, Sakyo, Kyoto Pref., 24-V-1991 (as larva on *Asarumasperum*), emerged on ?-VII-1991.

Ranunculaceae: • 2 adults, Shiriya, Higashidôri, Shimokita, Aomori Pref., 16-VI-1995 (as larva on *Aconitumjaponicum*), emerged on 13–14-VII-1995 (Fig. 11I–M); • 6 adults, Mt. Teine, Sapporo, Hokkaidô, 10-VII-1995 (as larva on *Aconitumyezoense*), emerged on 5-VIII-1995; • 4 adults, Monbetsu, Hidaka, Saru-gun, Hokkaidô, 5-VI-1993 on *Aconitumyezoense*; 3 adults, Mt. Obira, Shimamaki, Hokkaidô, 6-VII-2011 (as larva on *Aconitumpterocaule*, emerged on 28-VII-2011; • 5 adults, Hinoemata, Aizu-gun, Fukushima Pref., 16-VII-2023 (as larva on *Aconitumjaponicum*, emerged on 17–21-VIII-2023; 5 adults, Mt. Hiuchi, Myôkô, Niigata Pref., 16-VII-2023 (as larva on *Aconitumjaponicum*), emerged on 17–21-VIII-2023 (Fig. 11N); 12 adults, Funakawa, Oga, Akita Pref., 2-VI-2017 (as larva on *Aconitumjaponicum*), emerged on 24–28-VII-2017; • 3 adults, Atsumi, Tsuruoka, Yamagata Pref., 2-VI-2017 (as larva on *Aconitumokuyamae*), emerged on 20-VII-2017; • 4 adults, Tokoro, Abashiri, Hokkaidô, 24-VII-2017 (as larva on *Aconitumgigas*), emerged on 3–15-IX-2017; • 1 adult, Mt. Yudono, Tsuruoka, Yamagata Pref., 28-VIII-2017 (as larva on *Aconitumpterocaule*), emerged on 15-X-2017; • 1 adult, Donden, Sado Is., Niigata Pref., 13-VII-2019 (as larva on *Aconitumjaponicum*), emerged on ?-XI-2019; • 2 adults, Mt. Fuji, Fujinomiya, Shizuoka Pref., 27-VI-2001 (as larva on *Aconitumjaponicum*), emerged on 9–12-VIII-2001.

Asteraceae: 14 adults, Aibetsu, Kamikawa, Hokkaidô, 26-VI-2016 (as larva on *Cirsiumkamtschaticum*), emerged on 5–21-VIII-1995 (Fig. 11O–S); 4 adults, Kashiwadai, Chitose, Hokkaidô, 26-VI-2017 (as larva on *C.kamtschaticum*), emerged on 8–15-VIII-2017; 3 adults, Rebun Is., Hokkaidô, 9-VII-1995 (as larva on *C.kamtschaticum*), emerged on 10-VIII-1995; 1 adult, Tôbai, Nemuro, Hokkaidô, 23-VII-2018 (as larva on *C.iito-kojianum*), emerged on 28-VIII-2018; 2 adults, Mt. Yûbari, Yubari, Hokkaidô, 20-VII-2020 (as larva on *C.austrohidakaense*), emerged on 5–9-IX-2020; 1 adult, Shiriya, Higashidôri, Shimokita, Aomori Pref., 16-VI-2017 (as larva on *C.aomorense*), emerged on 13-VII-2017; 1 adult, Funakawa, Oga, Akita Pref., 2-VI-2017 (as larva on *C.makinoi*), emerged on ?-VII-2017; 4 adults, Mt. Haguro, Tsuruoka, Yamagata Pref., 9-VII-2018 (as larva on *C.ugoense*), emerged on 10-VIII--2018; 1 adult, Mt. Hiuchi, Myôkô, Niigata Pref., 14-VIII-2023 (as larva on *C.otayae*), emerged on 10–11-IX-2023; 1 adult, Mt. Hiuchi, Myoko, Niigata Pref., 4-V-2002 (as larva on *C.oligophyllum*), emerged on 10-VI-2002; 2 adults, Iwakura, Sakyo, Kyoto Pref., 1-V-1999 (as larva on *C.kiotoense*), emerged on 4-VI-1999; 1 adult, Mt. Kiyosumi, Chiba Pref., 14-V-2008 (as larva on *C.japonicum*), emerged on 20-VII-2008; 2 adults, Tochiu, Takashima, Shiga Pref., 6-V-1998 (as larva on *C.yoshinoi*), emerged on 7-VI-2017; 1 adult, Ippekiko Lake, Ito, Shizuoka Pref., 15-V-1999 (as larva on *C.microspicatum*), emerged on 14-VI-1999; 2 adults, Mt. Mikusa, Katô, Hyôgo Pref., 20-V-2018 (as larva on *C.japonicum*), emerged on 27–28-VI-2018; 1 adult, Yasukawa-keikoku, Tanabe, Wakayama Pref., 15-IV-2007 (as larva on *C.yoshinoi*), emerged on 16-V-2007; 8 adults, Hashirajima Is. Iwakuni, Yamaguchi Pref., 8-V-1993 (as larva on *C.japonicum*), emerged on 14–17-VI-1993.

#### ﻿ Argopus nigripennis

Taxon classificationAnimaliaColeopteraChrysomelidae

(Gebler, 1823)

14D48865-94EC-5568-A57B-656FC7F3C347

##### Note.

A host plant has not been reported (Hayashi et al. 1984; Kimoto and Takizawa 1993).

#### ﻿ Argopus unicolor

Taxon classificationAnimaliaColeopteraChrysomelidae

Motschulsky, 1860

8D694144-8AE0-5F85-A942-512701F68B15

##### Note.

A host plant has not been reported (Hayashi et al. 1984; Kimoto and Takizawa 1993).

### ﻿*Sphaeroderma* Stephens, 1831

#### ﻿ Sphaeroderma nigricolle

Taxon classificationAnimaliaColeopteraChrysomelidae

Jacoby, 1885

E3542A3C-C800-51BF-BD26-8046AC0F4712

Fig. 12A–O

##### Host plant.

Liliaceae: *Cardiocrinumcordatum* (Thunb.), *Liliumauratum* Lindley, *Tricyrtisflava* Maxim, *T.macropoda* Miq.; Smilacaceae: *Smilaxchina* L., *S.nipponica* Miq., *S.riparia* A. DC., *S.stans* Maxim., *Heterosmilaxjaponica* Kunth; Stemonaceae: *Croomiaheterosepala* (Baker), *C.japonica* Miq. Note: this species was collected on 11 host plants (six genera and three families of monocots, and the emerged adults varied in color and size among their host plants).

##### Leaf mine.

**Figure 12.**
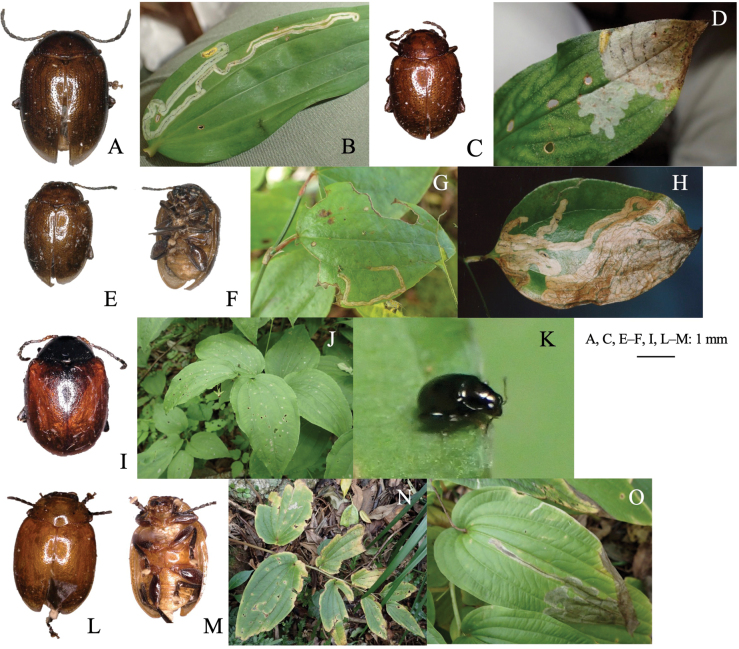
Habitus of adults and leaf mines of host races of *Sphaerodermanigricolle*. Host plants **A, B***Liliumauratum* at Mt. Haguro-san, Yamagata Pref. **C, D***Tricyrtismacropoda* at Tengu-kogen, Kôchi Pref. **E–G***Smilaxstans* at Mt. Torigata, Kôchi Pref. **H***Smilaxstans* at Mt. Ôtaki, Kagawa Pref. **I–K***Croomiaheterosepala* at Kikuchi-keikoku, Kumamoto Pref. **L–O***Croomiajaponica* at Naon, Amami-ôshima Is., Kagoshima Pref.

Full-depth long linear mine on mature leaf, often adjoining its own previous trajectory (Fig. 12B, D, G, H, J, N. O). Frass is thread-like, deposited along the middle line of the mine. The fully grown larva exits the mined leaf, falls to the ground, and pupates underground. The mine on *Tricyrtis* is unique in that the mine in young instars is not linear but radiate (Fig. 12D).

##### Material examined.

• 5 adults, Mt. Haguro, Tsuruoka, Yamagata Pref., 8-VII-2018 (as larva on *Liliumauratum*), emerged on 31-VIII–7-IX-2018 (Fig. 12A, B); • 1 adult, Iwakura, Sakyo, Kyoto Pref., 8-IX-2008 (as larva on *Tricyrtismacropoda*), emerged on 16-XI-2008 (Fig. 12C, D); • 1 adult, Mt. Torigata, Niyodogawa, Agawa-gun, Kôchi Pref., 6-X-2020 (as larva on *Smilaxstans*), emerged on 4-III-2021 (Fig. 9E–G); • 2 adults, Mt. Haguro, Tsuruoka, Yamagata Pref., 12-VI-2019 (as larva on *Smilaxnipponica*), emerged on 16-VIII-2019; several leaf mines, Mt. Ôtaki, Kagawa, Pref., 7-IX-1998 on *Smilaxstans* (Fig. 12H); • 1 adult, Kikuchi-keikoku, Kumamoto Pref., 2-V-2018 on *Croomiajaponica* (Fig. 12I–K); • 1 adult, Naon, Amami-ôshima Is., Kagoshima Pref., 12-XII-2014 (as larva on *Croomiaheterosepala*), emerged on 29-VI-2015 (Fig. 12L–O).

#### ﻿ Sphaeroderma japanum

Taxon classificationAnimaliaColeopteraChrysomelidae

Baly, 1874

93A02546-038B-5996-B8CD-D597C5387176

Fig. 13A–C

##### Host plant.

Commelinaceae: *Commelinacommunis* L.

##### Leaf mine.

**Figure 13.**
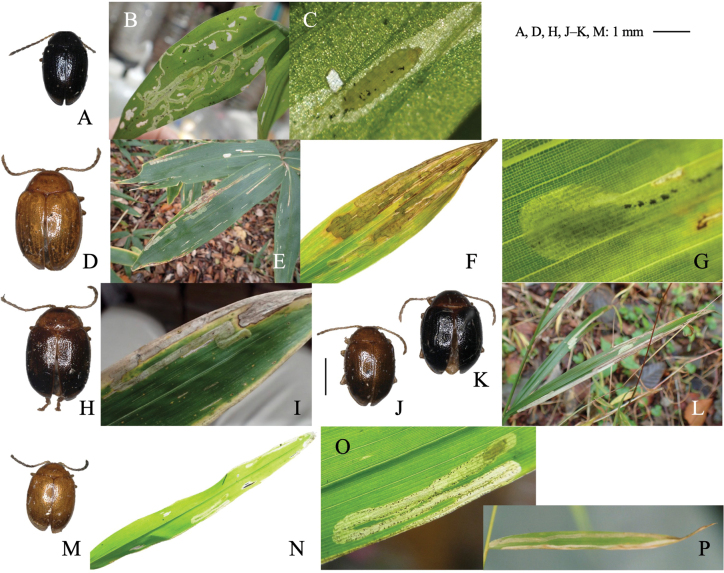
Habitus of adults and leaf mines of monocot-associated *Sphaeroderma* species **A–C***S.japanum***D–L***S.tarsatum***M–P***S.seriatum.* Host plants **A–C***Commelinacommunis* at Makidô, Niimi, Okayama Pref. **E–G***Sasanipponica* at Akiu, Sendai, Miyagi Pref. **H, I***Sasasenanensis* at Kamikôchi, Nagano Pref. **J–L**Stipacoreanavar.japonica at Mt. Fuji, Shizuoka **M–P***Panicumbisulcatum* at Yogo Lake, Shiga Pref. (**M–O**) and at Seryo, Kyôto Pref. (**P**).

Full-depth long linear mine on mature leaf (Fig. 13B, C). Frass is thread-like, deposited along the middle line of the mine. The fully grown larva exits the mined leaf, falls to the ground, and pupates underground.

##### Material examined.

• 5 adults, Maki-dô, Niimi, Okayama Pref., 22-VI-2020 (as larva on *Commelinacommunis*), emerged on 16-III-2021 (Fig. 13A–C).

#### ﻿ Sphaeroderma tarsatum

Taxon classificationAnimaliaColeopteraChrysomelidae

Baly, 1874

2043D58A-4285-579F-BDE8-A53696A667CE

Fig. 13D–L

##### Host plant.

Poaceae: *Phyllostachysbambusoides* Sieb. et Zucc., Pleioblastuschino(Franch. et Savat.)var.viridis (Makino), *Sasakurilensis* (Ruprecht), *S.nipponica* M. et S., *S.senanensis* (Franch et Savat.), *Sasamorphaborealis* (Hack.), *Shibataeakumasaca* Makino, StipacoreanaHondavar.kengii Ohwi).

##### Leaf mine.

Upper-layer long linear mine on mature leaf, often adjoining its own trajectory (Fig. 13E–G, I, L). The egg is laid in leaf blade, hatched larva mines usually toward leaf apex along leaf vein, and sometimes turns adjoining its past mine. Frass is granular, deposited linearly along the middle line of the mine. The mining larva is found in autumn from October to December. The fully grown larva exits the mined leaf from late autumn to early winter, falls to the ground, and pupates underground. Pupa hibernates under the ground, and the adult emerges the next spring.

##### Material examined.

• 2 adults, Akiu, Taihaku-ku, Sendai, Miyagi Pref., 14-XI-2014 (as larva on *Sasanipponica*), emerged on 7-V-2015 (Fig. 13D–G); • 1 adult, Azusagawa, Matsumoto, Nagano Pref., 24-X-2020 (as larva on *Sasasenanensis*), emerged on 2-III-2021 (Fig. 13H, I); • 1 adult, Ashiu, Nantan, Kyoto Pref., 9-XI-1999 (as larva on *Sasakurilensis*), emerged on 28-III-2000; 1 adult, Kuchisakamoto, Aoi-ku, Shizuoka, Shizuoka Pref., 27-XII-2005 (as larva on *Sasamorphaborealis*), emerged on 1-III-2006; • 1 adult, Inogashira, Fujinomiya, Shizuoka Pref., 11-XI-2001 (as larva on *Phyllostachysbambusoides*), emerged on 14-V-2002; • 3 adult, Usuzuka, Mt. Fuji, Fujinomiya, Shizuoka Pref., 24-X-2019 (as larva on Stipacoreanavar.kengii), emerged on 7–10-IV-2020 (Fig. 13J–L).

#### ﻿ Sphaeroderma seriatum

Taxon classificationAnimaliaColeopteraChrysomelidae

Baly, 1874

C6A481CE-3560-52CF-92CB-38D28E7F7745

Fig. 13M–P

##### Host plant.

Poaceae: *Panicumbisulcatum* Thunb.

##### Leaf mine.

Full-depth linear mine on mature leaf (Fig. 13N–P). The egg is laid along the leaf margin, and the hatched larva mines usually toward leaf apex along leaf margin, and sometimes turns adjoining its mine. Frass is intermittent thread-like, deposited linearly in two rows along both sides of the mine. Mining larva is found in October. The fully grown larva exits the mined leaf from late autumn to early winter, falls to the ground, pupates underground, and the adult emerges in late autumn.

##### Material examined.

• 3 adults, Yogo Lake, Nagahama, Shiga Pref., 12-IX-2015 (as larva on *Panicumbisulcatum*), emerged on 7-X-2015 (Fig. 13M–O); • 4 adults, Seryô, Sakyô, Kyoto, Kyoto Pref., 11-IX-1998 (as larva on *Panicumbisulcatum*), emerged on 28-IX–1-X-1998 (Fig. 13P).

#### ﻿ Sphaeroderma apicale

Taxon classificationAnimaliaColeopteraChrysomelidae

Baly, 1874

86AEF7ED-D0D2-552C-B3DD-C12315A26B89

##### Host plant.

Poaceae: *Miscanthussinensis* (Hayashi et al. 1984; Kimoto and Takizawa 1993).

##### Leaf mine.

Unknown.

#### ﻿ Sphaeroderma akebia

Taxon classificationAnimaliaColeopteraChrysomelidae

Ohno, 1964

7CB09DD1-636E-5BB4-A6D1-913F0AD888F0

Fig. 14A–C

##### Host plant.

Lardizabalaceae: *Akebiatrifoliata* (Thunb.), *A.quinata* Decne.

##### Leaf mine.

**Figure 14.**
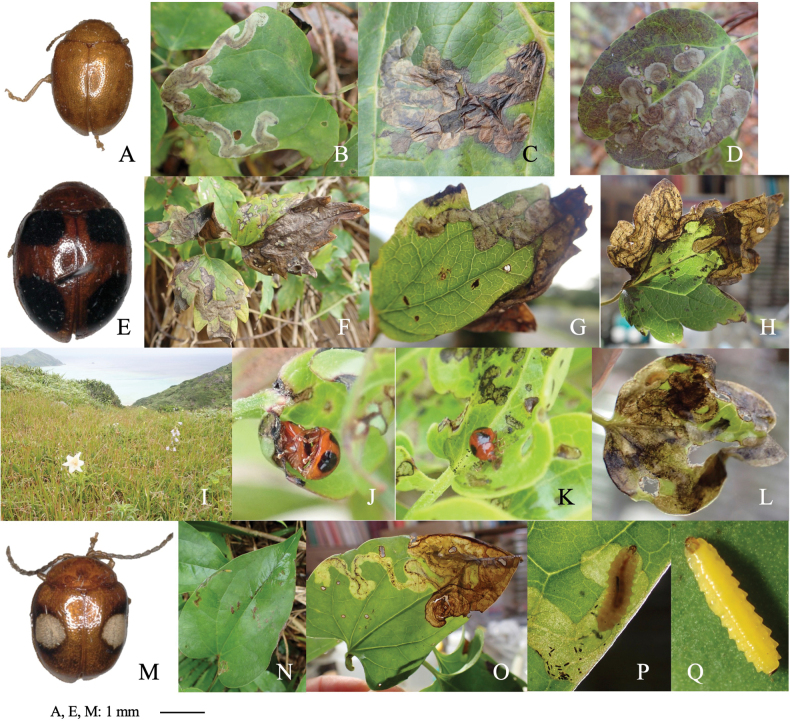
Habitus of adults, habitats, and leaf mines of *Akebia*-associated and *Clematis*-associated *Sphaeroderma* species **A–C***S.akebia***D***S.inaizumii***E–L***S.quadrimaculatum***M–Q***S.flavonotatum***R–V***S.separatum.* Host plants **A–C***Akebiatrifoliata* at Tsuruoka, Yamagata Pref. **D***Akebiatrifoliata* at Shôji Lake, Yamanashi Pref. **E–G**Clematistaiwanianavar.ryukiuensis at Hedo, Okinawa Is., Okinawa Pref. **J–L**Clematistaiwanianavar.ryukiuensis at Tonaki Is., Okinawa Pref. **M–P***Clematistashiroi* at Iriomote Is. Okinawa Pref. **S–V***Clematisjaponica* at Shirakawa-gô, Gifu Pref.

Upper-layer linear mine on mature leaf (Fig. 14B, C). The mine is wider than the larval width, having dead ends and branches. Frass is granular and minute, deposited linearly in two rows along both sides of the mine, and inner space between the frass lines is colored darker against the outer space. The mining larva is found from late autumn to early winter. The fully grown larva exits the mined leaf in early winter, falls to the ground, and pupates underground. The pupa hibernates underground, and the adult emerges the next spring.

##### Material examined.

• 3 adults, Shimoike Lake, Tsuruoka, Yamagata Pref., 15-XI-2014 (as larva on *Akebiatrifoliata*), emerged on 7-V-2015 (Fig. 14A–C); • 2 adults, Aibano, Takashima, Shiga Pref., 13-XII-1998 (as larva on *Akebiatrifoliata*), emerged on 8-IV-1999.

#### ﻿ Sphaeroderma inaizumii

Taxon classificationAnimaliaColeopteraChrysomelidae

Takizawa, 2015

D46F349D-3261-5568-8F15-8275E18C838F

Fig. 14D

##### Host plant.

Lardizabalaceae: *Akebiatrifoliata*, *A.quinata*.

##### Leaf mine.

Upper-layer blotch mine on mature leaf (Fig. 14D). The mine is orbicular, and the larva often relocates its mine. Frass is minute and often liquid, deposited as dark band in the mine. The mining larva is found from late autumn to early winter. The fully grown larva exits the mined leaf in early winter, falls to the ground, and pupates underground.

##### Material examined.

Many leaf mines, Shoji Lake, Kawaguchiko, Yamanashi Pref., 24-XI-2018 on *Akebiatrifoliata* (Fig. 14D).

#### ﻿ Sphaeroderma quadrimaculatum

Taxon classificationAnimaliaColeopteraChrysomelidae

Chûjô, 1935

0A2B5252-B029-52B8-AC5E-F3DDBD44C4A8

Fig. 14E–L

##### Host plant.

Ranunculaceae: ClematistaiwanianaHayatavar.ryukiuensis Tamura.

##### Leaf mine.

Full-depth linear mine on mature leaf (Fig. 14F–H, L). Frass is granular and minute, deposited linearly in two rows along both sides of the mine. The fully grown larva exits the mined leaf in early winter, falls to the ground, and pupates underground.

##### Material examined.

• 2 adults, Hedo, Kunigami, Okinawa Pref., 10-XI-2021 on Clematistaiwanianavar.ryukiuensis (Fig. 14E); • 3 adults, Hedo, Kunigami, Okinawa Pref., 14-X-2000 (as larva on C.taiwanianavar.ryukiuensis), emerged on 21-XI–10-XII-2000 (Fig. 14F–H); • 2 adults, Tonaki Is., Shimajiri-gun, Okinawa Pref., 18-III-2020 (as larva on C.taiwanianavar.ryukiuensis), emerged on 10-V-2020 (Fig. 14I–L).

#### ﻿ Sphaeroderma flavonotatum

Taxon classificationAnimaliaColeopteraChrysomelidae

Chûjô, 1937

D7143DFD-E8E2-5947-B5F7-A94FFA847433

Fig. 14M–Q

##### Host plant.

Ranunculaceae: *Clematistashiroi* Maxim. The host plant record of *Smilax* spp. by Takizawa (2021) is uncertain.

##### Leaf mine.

Full-depth linear mine on mature leaf (Fig. 14N–P). Frass is granular, deposited as a band along middle line of the mine. The fully grown larva (Fig. 14Q) exits the mined leaf in early winter, falls to the ground, and pupates underground.

##### Material examined.

• 6 adults, Funaura, Iriomote Is., Yaeyama, Okinawa Pref., 6-III-2019 (as larva on *Clematistashiroi*), emerged on 20-IV-2019 (Fig. 14M–Q).

#### ﻿ Sphaeroderma separatum

Taxon classificationAnimaliaColeopteraChrysomelidae

Baly, 1874

B91FA7A4-9F9E-5B8F-9F75-9E8DE1B58AD9

Fig. 14R–V

##### Host plant.

Ranunculaceae: *Clematisjaponica*. Takizawa (2021) lists up three host plants: *C.apiifolia* DC., *C.pierotti* Miq. and *Chelidoniummajus* L. (Papaveraceae).

##### Leaf mine.

Full-depth radiate mine along leaf vein on mature leaf (Fig. 14T–V). Frass is liquefied, deposited near the center of the mine.

##### Material examined.

• 2 adults copulating on a leaf of *Clematisjaponica* at Shirakawa-gô, Shirakawa, Ôno-gun, Gifu Pref., 12-VIII-2024 (Fig. 14R, S), and many leaf mines on the plant species at the same locality 29-IX-2023 (Fig. 14T–V).

#### ﻿ Sphaeroderma placidum

Taxon classificationAnimaliaColeopteraChrysomelidae

Harold, 1877

CC7B707B-EFDD-5CFD-9639-76061E7E27E4

Fig. 15A–G

##### Host plant.

Ranunculaceae: *Clematisapiifolia*.

##### Leaf mine.

Black upper-layer blotch mine on mature leaf. Frass is granular scattered along larval trajectory at young instars, and accumulated in a discoid area in a center of the mine in old instars. The mining larva is found from late autumn to early winter. The fully grown larva exits the mined leaf in early winter, falls to the ground, and pupates underground. The pupa hibernates under the ground, and the adult emerges the next spring.

##### Material examined.

**Figure 15.**
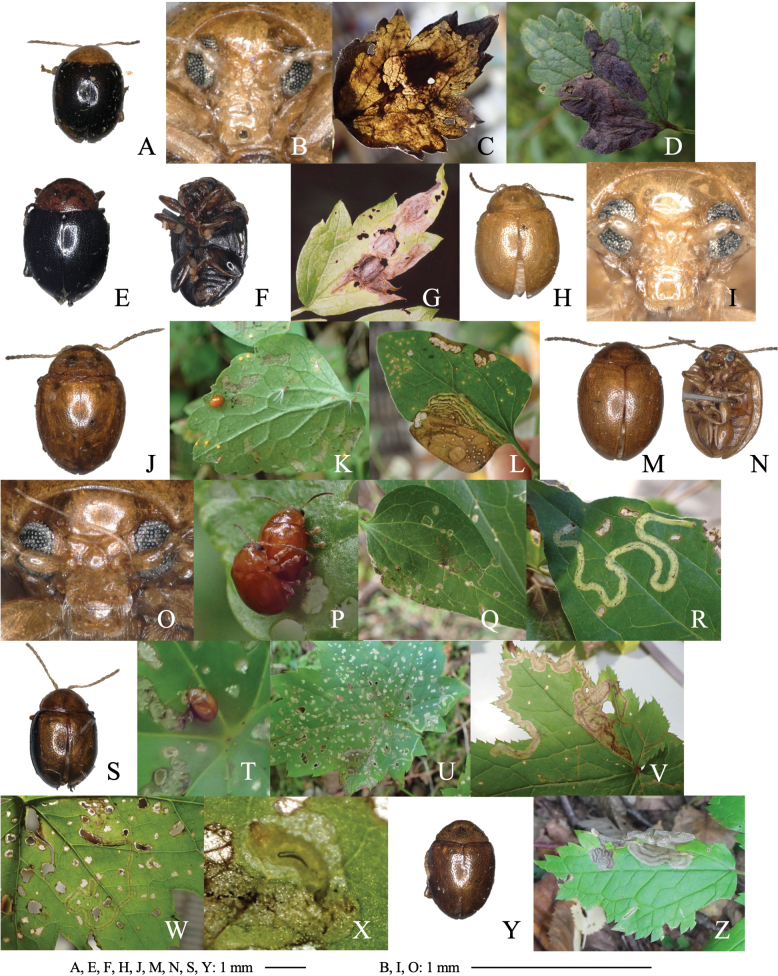
Adult morphology, behavior, and leaf mines of *Clematis*-associated and *Cimicifuga*-associated *Sphaeroderma* species **A–G***S.placidum***H–L***S.unicolor***M–R***S.uenoi***S–Z***S.ohkuboi***A, E, H, J, M, S, Y** adult habitus in dorsal view **F, N** ventral view **B, F, I, O** Frontal view of head **K, P, T** adult feeding behavior **C, D, G, L, Q, R, U–X, Z** leaf mines. Host plants **A–D***Clematisapiifolia* at Shyôji Lake, Yamanashi Pref. **E–G***Clematisapiifolia* at Ashiu, Kyôto Pref. **H, I***Clematisterniflora* at Obama, Fukui Prefecture **J–L***Clematisterniflora* at Iya, Tokushima Prefecture **M–Q***Clematisterniflora* at Kizu-gawa, Kyôto Pref. **R***Clematisterniflora* at Iwakura, Kyôto Pref. **S–X***Cimicifugajaponica* at Mt. Nakatsu-myôjin, Kôchi Pref. **Y, Z***Cimicifugasimplex* at Shirahone-onsen, Nagano Pref.

• 3 adults, Shoji Lake, Kawaguchiko, Yamanashi Pref., 24-XI-2018 (as larva on *Clematisapiifolia*), emerged on 10-V-2019 (Fig. 15A–D); • 2 adults, Kisofukushima, Kiso, Nagano Pref., 24-X-1999 (as larva on *Clematisapiifolia*), emerged on 22-V-2000; 11 adults and several leaf mines, Fukasawa, Gotenba, Shizuoka Pref., 29-VI-2014 on *Clematisapiifolia*; • 1 adult and several leaf mines on *Clematisapiifolia*, Ashiu, Nantan, Kyôto Pref., 13-IX-1993 (Fig. 15E–G).

#### ﻿ Sphaeroderma unicolor

Taxon classificationAnimaliaColeopteraChrysomelidae

Kimoto, 1965

E8BC6C9E-138E-585C-8D5C-1EF1359589DA

Fig. 15H–L

##### Host plant.

Ranunculaceae: *Clematisterniflora* DC., *C.apiifolia* DC.

##### Leaf mine.

Full-depth linear mine on mature leaf, often adjoining its own trajectory (Fig. 15L). Frass is linear thread-like, deposited along a side line of the mine. Mining larva is found from late autumn to early winter. The fully grown larva exits the mined leaf in early winter, falls to the ground, and pupates underground. The pupa hibernates under the ground, and the adult emerges the next spring.

##### Material examined.

• 1 adult and several leaf mines, Iya, Miyoshi, Tokushima Pref., 13-VI-2017 on *Clematisapiifolia* (Fig. 15J–L); • 5 adults, Aidani, Iwade, Wakayama Pref., 19-I-2002 (as larva on *Clematisterniflora*), emerged on 28-III–1-IV-2002; • 1 adult, Inogashira, Fujinomiya, Shizuoka Pref., 11-XI-1999 (as larva on *Clematisterniflora* collected by T. Kato), emerged on 14-V-2000; • 4 adults, Obama, Fukui Pref., 13-XII-1998 (as larva on *Clematisterniflora*) emerged on 4–8-IV-1998 (Fig. 15H, I).

#### ﻿ Sphaeroderma uenoi

Taxon classificationAnimaliaColeopteraChrysomelidae

Takizawa, 2021

6C1FB0F6-B295-5518-A89C-C7168BC8900D

Fig. 15M–R

##### Host plant.

Ranunculaceae: *Clematisapiifolia*, *Clematisterniflora*.

##### Leaf mine.

Full-depth linear mine on mature leaf (Fig. 15R). Frass is linear thread-like but intermittent. The fully grown larva exits the mined leaf in early winter, falls to the ground, and pupates underground. The pupa hibernates under the ground, and the adult emerges the next spring.

##### Material examined.

• 2 adults and several leaf mines on *Clematisterniflora*, Kizu-gawa, Kusauchi, Kyôtanabe, Kyoto Pref., 11-X-2022 (Fig. 15M–Q); many leaf mines on *Clematisterniflora*, Iwakura, Sakyo, Kyoto Pref., 20-XII-2023 (Fig. 15R).

#### ﻿ Sphaeroderma ohkuboi

Taxon classificationAnimaliaColeopteraChrysomelidae

Chûjô, 1940

5580FCC1-C832-58DA-BB2B-0B6D2D19DD2F

Fig. 15S–Z

##### Host plant.

Ranunculaceae: *Cimicifugajaponica* (Thunb.), *C.biternata* (Siebold et Zucc.), *C.simplex* Wormsk.

##### Leaf mine.

Full-depth linear mine on the mature leaf (Fig. 15V–X, Z). Frass is dark pasty, deposited as a wide band along middle line of the mine. The fully grown larva exits the mined leaf in autumn, falls to the ground, and pupates underground. The pupa hibernates under the ground, and the adult emerges the next spring.

##### Material examined.

• 1 adult and several leaf mines, Mt. Nakatsu-myôjin, Niyodogawa, Kôchi Pref., 6-X-2020 on *Cimicifugajaponica* (Fig. 15S–X); 1 adult, Shirahone, Matsumoto, Nagano Pref., 15-X-2013 (as larva on *C.simplex*), emerged on ?-V-2014 (Fig. 15Y, Z); • 1 adult, Toyohara, Nasu, Tochigi Pref., 14-IX-2003 (as larva on *C.simplex*), emerged on 3-V-2004.

#### ﻿ Sphaeroderma balyi

Taxon classificationAnimaliaColeopteraChrysomelidae

Jacoby, 1885

F90151A6-EA30-5851-A57A-E69B16CE0006

Fig. 16A–J

##### Host plant.

Asteraceae: *Farfugiumjaponicum* (L.), *Paraseneciokamtschaticus* (Maxim.), *P.amagiensis* (Kitam.), *P.yatabei* (Matsum. et Koidz.), *Petasitesjaponicus* (Siebold et Zucc.).

##### Leaf mine.

**Figure 16.**
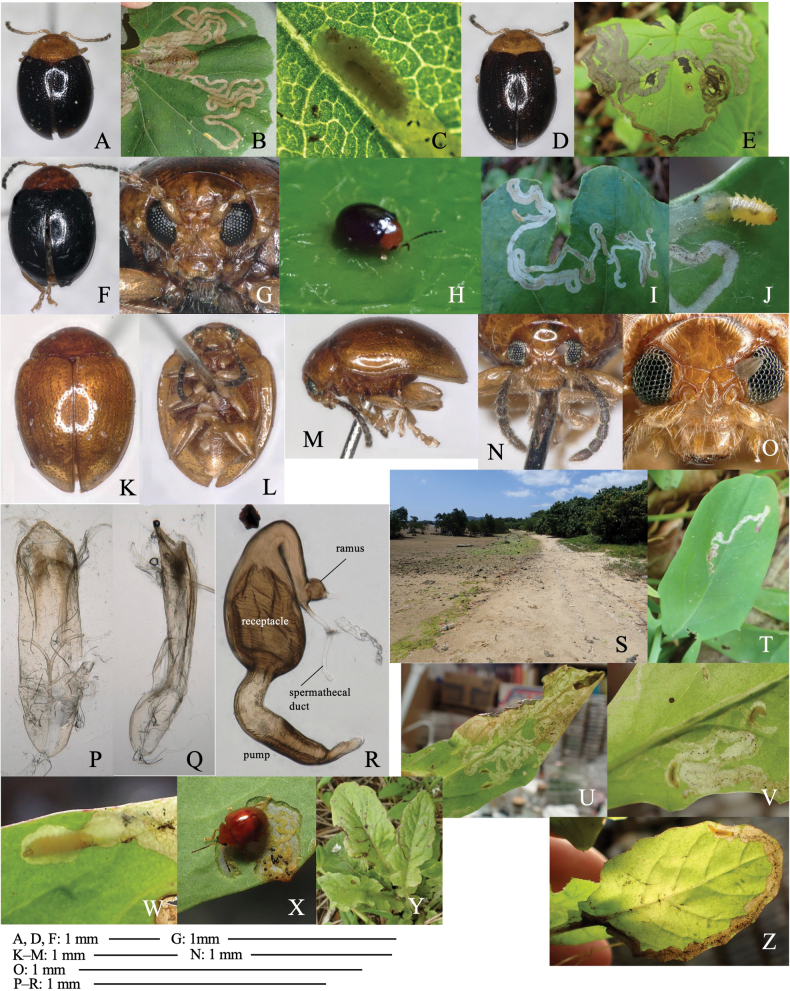
Adult morphology, behavior, and leaf mines of Asteraceae-associated *Sphaeroderma* species **A–J***S.balyi***K–Z***S.komiana* sp. nov. **A, D, F** adult habitus in dorsal view **G, O** frontal view of head **H, X** adult feeding behavior **L–N** ventral, lateral, frontal view **P–Q** male genitalia **R** female spermatheca **S** landscape of the habitat at type locality **B, C, E, I, J, T–W, Y, Z** leaf mines. Host plants **A–C***Petasitesjaponicus* at Ukawa, Kyôtango, Kyôto Pref. **D, E***Paraseneciokamtschaticus* at Tôbai, Nemuro, Hokkaido **F–J***Farfugiumjaponicum* at Kobukei, Nichinan, Miyazaki Pref. **T–X***Ixerisjaponica* at Komi, Iriomote Island, Okinawa Pref. **Y–Z***Youngiajaponica* at Hoshidate, Iriomote Is., Okinawa Pref.

Upper-layer linear mine on the mature leaf, with trajectories usually adjoining each other (Fig. 16B, E, I, J). Frass is minute granular, deposited linearly along either side of the mine. The fully grown larva exits the mined leaf in autumn, falls to the ground, and pupates underground.

##### Material examined.

• 2 adults, Mt. Teine, Sapporo, Hokkaidô, 10-VII-1995 (as larva on *Petasitesjaponicus*), emerged on 10-VIII-1995; • 1 adult, Shimoike, Tsuruoka, Yamagata Pref., 12-VI-2019 (as larva on *Petasitesjaponicus*), emerged on 10-VII-2019; • 1 adult, Mt. Haguro, Tsuruoka, Yamagata Pref., 8-VII-2018 (as larva on *Petasitesjaponicus*), emerged on ?-IV-2019; 1 adult, Renge-onsen, Itoigawa, Niigata Pref., 1-VII-2013 (as larva on *Petasitesjaponicus*), emerged on 10-VIII-2013; • 4 adults, Ukawa, Kyôtango, Kyoto Pref., 17-IX-2019 (as larva on *Petasitesjaponicus*), emerged on 15–20-X-2019 (Fig. 16A–C); • 1 adult, Nagawado, Matsumoto, Nagano Pref., 12-VII-2003 (as larva on *Petasitesjaponicus*), emerged on 1-IX-2003; • 2 adults, Tôbai, Nemuro, Hokkaidô, 23-VII-2018 (as larva on *Paraseneciokamtschaticus*), emerged on 30-VII–4-VIII-2018 (Fig. 16D, E); • 1 adult, Torikura-rindo, Ôshika, Nagano Pref., 30-VI-2013 (as larva on *Parasenecioyatabei*), emerged on 28-VIII-2013; • 1 adult, Yawatano, Izu, Shizuoka Pref., 12-V-2002 (as larva on *Parasenecioamagiensis* collected by T. Kato), emerged on 26-VI-2002; • 3 adults, Amatsu-kominato, Kamogawa, Chiba Pref., 14-V-2008 (as larva on *Farfugiumjaponicum*), emerged on 27-V-2008; 3 Dôgashima, Nishiizu, Shizuoka Pref., 9-V-2004 (as larva on *Farfugiumjaponicum*), emerged on 23-VI-2004; 3 Ena, Yura, Hidaka-gun, Wakayama Pref., 19-V-2002 (as larva on *Farfugiumjaponicum*), emerged on 12–15-VI-2002; • 2 adults, Ashizuri-misaki, Tosashimizu, Kochi Pref., 23-IV-2018 (as larva on *Farfugiumjaponicum*), emerged on 7-VI-2018; • 1 adult and manly leaf mines, Kobukei, Nichinan, Miyazaki Pref., 23-X-2022 on *Farfugiumjaponicum* (Fig. 16F–J).

#### ﻿ Sphaeroderma fulvoapicale

Taxon classificationAnimaliaColeopteraChrysomelidae

Kimoto & Gressitt, 1966

1F853C6B-AEB5-5C94-BE52-BA058DC058BB

##### Host plant.

Asteraceae: *Farfugiumjaponicum* (L.) (Takizawa, 2021).

##### Leaf mine.

Unknown.

#### ﻿ Sphaeroderma komiana

Taxon classificationAnimaliaColeopteraChrysomelidae

Kato sp. nov.

F579D9DA-E514-5EA1-86C7-552BCB3E8B73

https://zoobank.org/390A08F4-3F79-4548-8F94-409EA1A23E5A

Fig. 16K–Z

##### Type locality.

Japan: Okinawa Pref., Iriomote Is., Komi.

##### Type material.

***Holotype***: • ♂, Komi, Iriomote Is., Yaeyama, Okinawa Pref. (24.319°N, 123.910°E, 4 m above sea level), 16-III-2018 (collected as larva on *Ixerisjaponica* by M. Kato), emerged on 15-V-2018 (NSMT-I-C- 200350). ***Paratypes***: • 3 ♂ 1 ♀, same data with holotype, emerged on 11–15-V-2018 (NSMT-I-C- 200351–200354); • 3 ♂ 2 ♀, Shirahama, Iriomote Is., Yaeyama, Okinawa Pref., 16-III-1999 (as larva on *Lactucaindica*), emerged on 21–25-IV-1999 (NSMT-I-C-200355– 200359).

##### Diagnosis.

The species is a small oblong-oval, strongly convex beetle (length 1.8–1.9 mm) with a shiny, completely reddish brown body, elytra, and legs. The head features a pair of distinctly delimited frontal tubercles that contact each other at postero-inner angles. The inter-antennal area is raised and fusiform. The male genitalia exhibit a laterally uncurved aedeagus. The larva mines the leaves of Asteraceae plants including *Ixerisjaponica*, *Lactucaindica*, and *Youngiajaponica*.

##### Description.

**Adult male** (Fig. 16K–Q). ***Habitus*.** The body is oblong-oval and strongly convex on the dorsal side, measuring 1.8–1.9 mm in length (Fig. 16K, N). It is reddish brown, with black eyes. The antennae are dark brown, and the four basal segments, pronotum, and elytra are reddish brown (Fig. 16N).

***Head*.** The head has a smooth, shiny, impunctate vertex. The frontal tubercles are transverse and posteriorly delimited by a nearly straight, deep, sharp sulcus, with antero-inner and antero-outer angles produced below, well-delimited behind by a sharp furrow, almost contacting each other at postero-inner angles (Fig. 16O). The inter-antennal area is raised and fusiform, with the diameter of the raised area narrower than that of the antennal socket. The eyes are strongly convex, with their transverse diameter in frontal view being 0.8-fold wider than the inter-ocular distance. The clypeus has an entire anterior fringe. The antennae are half as long as the body. The proportional lengths of antennomeres 1–11 are as follows: 1:0.50:0.38:0.51:0.65:0.65:0.65:0.65:0.69:0.69:0.91.

***Thorax*.** The pronotum is transverse, 1.7-fold as wide as long, with the widest point located slightly before basal angles, and broadly arched at the posterior margin, with roundly produced anterior angles. The disc is evenly convex, sparsely covered with small punctures and interspaced with smooth and shining areas. The scutellum is rounded and triangular in shape, flat, impunctate, and as long as wide. The elytra are oblong and strongly convex, each measuring 2.1-fold as long as wide, widest at the basal one-fourth area and then rounded and narrowed toward the apex (Fig. 16K). The disc is densely covered with 11 partially irregular, longitudinal striae of small punctures. The epipleura are wide at the base, with gradual narrowing and disappearance before the apex. The epipleural disc is impunctate and smooth. The prosternum is narrow with a stout longitudinal carina as wide as the length of the 10^th^ antennal segment (Fig. 16L).

***Abdomen*.** The fifth visible abdominal sternite is densely covered with punctures bearing long hairs and is weakly concave apically, with a dark median longitudinal line. The legs are stout, with the first tarsal segments being moderately enlarged but distinctly narrower than the third segment. The hind legs have significantly enlarged femora.

***Genitalia*.** (Fig. 16P, Q) The aedeagus is lanceolate in dorsal view, 3.6-fold longer than its width, almost parallel-sided, and narrowed to a rounded triangular apex. Slightly curved in lateral view, with the ventral surface almost flat. The ostium is membranous, containing an inverted V-shaped sclerotized area.

**Female**. The body is slightly enlarged, ~ 2.0–2.2 mm in length.

***Genitalia*.** (Fig. 16R) The spermatheca is brown and sclerotized, consisting of a proximal swollen receptacle and a distal strongly curved slender pump, with the apex attenuated and curved inward. The receptacle and pump exhibit many transverse wrinkles. The spermathecal duct is proximally sclerotized, connecting to a thin transparent duct. The distal portion of the sclerotized spermathecal duct carries a globular ramus.

##### Distribution.

The only known distribution is on Iriomote Island, Japan.

##### Host plant.

Asteraceae: *Ixerisjaponica* (Burm. f.) (Fig. 16T–X), *Lactucaindica* L., *Youngiajaponica* (L.) (Fig. 16Y, Z). *Ixerisjaponica* grows on sandy beaches along coral reefs and mangroves (Fig. 16S). *Lactucaindica* and *Y.japonica* grow along margins of rice field paddies.

##### Leaf mine.

Full depth linear mines on mature leaf, without crossing, backtracking, or branching (Fig. 16T–W, Y, Z). Typically, the mine is initiated near the midrib, seldom adjoining other mines. Minute granular frass is linearly deposited along the subcentral line of the mine. Upon reaching maturity, the larva exits the mined leaf in autumn and drops to the ground, where it pupates beneath the soil surface.

##### Etymology.

The species name refers to the village name of the type locality, which is also the original name of Iriomote Island (Komi).

##### Japanese name.

Komi-tamanomi-hamushi.

##### Remarks.

This newly identified species resembles asterid-associated *S.balyi* in terms of size, habitus, punctuation of elytra, and frontal tubercles of the head. However, it is distinctly differentiated from the latter by the reddish-brown color of the elytra (black in *S.balyi*), parallel-sided and dorso-ventrally flattened aedeagus (aedeagus in the latter having lateral constriction in the middle and strongly curved in lateral view; Kimoto and Takizawa 1993: pl. 84, fig. 9), host plant genera (*Ixeris*, *Lactuca*, and *Youngia* vs *Farfugium*, *Parasenecio*, and *Petasites*), and mining pattern without adjoining trajectories (the mine of the latter is characterized by adjoining meandering trajectories; Fig. 16B, E, I).

This new species also resembles *Clematis*-associated *Sphaeroderma* species (*C.unicolor* and yellow form of *C.uenoi*) in terms of habitus and body color. However, it is distinguished from the latter by the darker color of elytra compared to their yellowish color in *C.unicolor* and yellow color in *C.uenoi*, a pair of frontal tubercles contacting each other compared to distinct tubercles in the latter (Takizawa 2021: figs 7, 10), and laterally almost uncurved aedeagus of the male genitalia compared to the significantly aedeagus in the latter (Takizawa 2021: figs 12, 15).

Fifteen species of *Sphaeroderma* have been recorded from Taiwan, 13 of which are endemic to the region (Kimoto and Takizawa 1997; Lee 2023). The newly identified species resembles *Sphaerodermahsui* Lee, 2023, a Taiwanese species, in terms of its habitus and small size. However, it can be differentiated from the latter based on its dark brown antennae compared to the yellowish-brown antennae in the latter, transverse frontal tubercle compared to the rounded or subsquare frontal tubercle in the latter, laterally uncurved aedeagus of male genitalia compared to the laterally moderately curved aedeagus in the latter, and a slender spermathecal pump with an apical tubular appendage compared to a short pump without apical appendage in the latter.

Among 21 *Sphaeroderma* species recorded from Japan (Takizawa 2021), the following five species have no known host plants (Hayashi et al. 1984; Kimoto and Takizawa 1993):

*Sphaerodermaatrum* Jacoby, 1885

*Sphaerodermakuroashi* Kimoto, 2000

*Sphaerodermamorimotoi* Chûjô & Ohno, 1964

*Sphaerodermaobscurum* Ohno, 1964

*Sphaerodermarubidum* (Graells, 1858)

### ﻿Cassidinae Gyllenhal, 1813

**Hispini Gyllenhal, 1813**

***Leptispa* Baly, 1858**

#### ﻿ Leptispa taguchii

Taxon classificationAnimaliaColeopteraChrysomelidae

Chûjô, 1956

80FEE769-2FBF-5F1D-90C2-B749BE88FF79

##### Host plant.

Poaceae: *Miscanthussinensis* Andersson (Hayashi et al. 1984; Kimoto and Takizawa 1993).

##### Leaf mine.

Unknown.

#### ﻿ Leptispa miyamotoi

Taxon classificationAnimaliaColeopteraChrysomelidae

Kimoto, 1957

5BC1ADE9-62A5-58D7-B623-D9995E5EA9B7

##### Host plant.

Poaceae: *Miscanthussinensis*, *Saccharumofficinarum* L. (Hayashi et al. 1984; Kimoto and Takizawa 1993).

##### Leaf mine.

Unknown.

### ﻿Asamangulia Maulik, 1915

#### ﻿ Asamangulia yonakuni

Taxon classificationAnimaliaColeopteraChrysomelidae

(Kimoto & Gressitt, 1966)

2613D0F4-B430-592D-9501-F49EA1966413

##### Host plant.

Poaceae: *Oryzasativa* L., *Miscanthussinensis* (Hayashi et al. 1984; Kimoto and Takizawa 1993).

##### Leaf mine.

Unknown.

### ﻿*Dactylispa* Weise, 1897

#### ﻿ Dactylispa subquadrata

Taxon classificationAnimaliaColeopteraChrysomelidae

(Baly, 1874)

F54BE6A9-D214-5229-AB89-EF02AC07ECEC

Fig. 17A–D

##### Host plant.

Fagaceae: *Castaneacrenata* Sieb. et Zucc., *Castanopsissieboldii* (Makino), *Quercusdentata* Thunb., *Q.aliena* Blume, *Q.serrata* Thunb., *Q.variabilis* Blume, *Q.glauca* (Thunb.).

##### Leaf mine.

**Figure 17.**
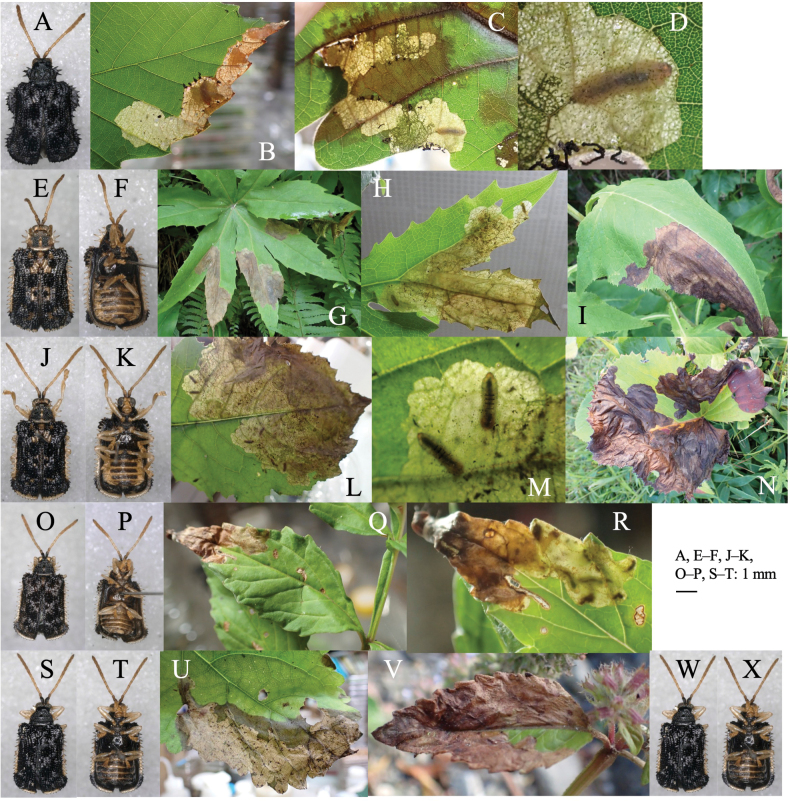
Habitus of adults and leaf mines of three *Dactylispa* species **A–D***D.subquadrata***E–N***D.masonii***O–X***D.angulosa*. Host plants **A, B***Quercusserrata* at Yashiro, Hyôgo Pref. **C, D***Quercusdentata* at Nasu, Tochigi Pref. **E–H***Syneilesispalmata* at Kawazu, Izu, Shizuoka Pref. **N–S**Paraseneciohastatussubsp.orientalis at Aikappu, Akkeshi, Hokkaido **J–M***Cirsiumsuzukaense* at Mt. Ibuki, Shiga Pref. **N***Ligulariahodgsonii* at Aikappu, Akkeshi, Hokkaido **O–R***Salviaranzaniana* at Sabushi-gawa, Niimi, Okayama Pref. **S–U***Salvianipponica* at Nasu, Tochigi Pref. **V***Clinopodiummicranthum* at Nagawado, Nagano Pref. **W–X***Lithospermumzollingeri* at Taishaku-kyô, Okayama Pref.

Full-depth linear-blotch mine on mature leaf (Fig. 17B–D). The mine has many holes perforated by larva on each side of the mine, and frass is excreted outside through the holes. The fully grown larva pupates in the mine. The upper layer tissue around the pupation site is often kept intact, so that the pupa is hidden by the green tissue and kept less conspicuous.

##### Material examined.

• 11 adults, Yashiro, Katô, Hyôgo Pref., 25-VI-2019 (as larva on *Quercusserrata*), emerged on 19–23-VII-2019 (Fig. 17A–C); Several leaf mines, Toyohara, Nasu, Tochigi Pref., 2-VII-2022 on *Quercusdentata* (Fig. 17D); • 1 adult, Hami, Miyazu, Kyoto Pref., 3-VIII-2008 (as larva on *Quercusvariabilis*), emerged on 4-VIII-2008; • 1 adult, Hami, Miyazu, Kyoto Pref., 16-VII-2012 (as larva on *Q.glauca*), emerged on 1-VIII-2012; • 1 adult, Kushimoto-ôshima, Higashimuro-gun, Wakayama Pref., 2-VIII-1999 (as larva on *Castanopsissieboldii*), emerged on 3-VIII-1999; • 1 adult, Hami, Miyazu, Kyoto Pref., 3-VIII-2008 (as larva on *Castaneacrenata*), emerged on 5-VIII-2008.

#### ﻿ Dactylispa masonii

Taxon classificationAnimaliaColeopteraChrysomelidae

Gestro, 1923

C1B6D22C-E9F2-56E5-BA36-AD3583FB7124

Fig. 17E–N

##### Host plant.

Asteraceae: *Ainsliaeaacerifolia* Sch. Bip., *Asteryomena* (Kitam.), CacaliaauriculataDC.var.kamtschatica (Kitam.), *Cirsiumashiuense* S. Yokoy. et T. Shimizu, *C.confertissimum* Nakai, *C.inundatum* Makino, *C.kiotoense*, *C.longepedunculatum* Kitam., *C.microspicatum* Nakai, *C.okamotoi* Kitam., *C.olygophyllum* (Franch. et Sav.), *C.sieboldii* Miq., *C.suzukaense* Kitam., C.tashiroiKitam.var.hidaense (Kitam.), *C.tonense* Nakai, *Ligulariafischeri* (Ledeb.), *L.hodgsonii* Hook., Paraseneciohastatus(L.)ssp.orientalis (Kitam.), P.farfarifolius(Siebold et Zucc.)var.bulbiferus (Maxim.), *Petasitesjaponicus*, *Syneilesispalmata* (Thunb.).

##### Leaf mine.

Dark upper-layer aggregate blotch mine on mature leaf (Fig. 17G–I, L–N). Frass is granular, deposited along meandering larval trajectory. Often several larvae aggregately mine in a leaf, and the mined area is large and turns blackish. The fully grown larvae pupate together in the mine.

##### Material examined.

• 20 adults, Kawazu, Kamo-gun, Shizuoka Pref., 7-VI-2015 (as larva on *Syneilesispalmata*), emerged on 15–20-VI-2015 (Fig. 17E–H); • 2 adults, Aikappu, Akkeshi, Hokkaidô, 4-VIII-2023 (as larva on Paraseneciohastatusssp.orientalis), emerged on 19-VIII-2023 (Fig. 17I); • 4 adults, Hotokegaura, Sai-mura, Shimokita, Aomori Pref., 27-VII-2009 (as larva on Paraseneciofarfarifoliusvar.bulbiferus), emerged on 24–28-VIII-2009; 1 adult, Renge-onsen, Itoigawa, Niigata Pref., 4-IX-1999 (as larva on *Ainsliaeaacerifolia*), emerged on 15-IX-1999; • 2 adults, Suehiro, Akkeshi, Hokkaidô, 4-VIII-2023 (as larva on *Ligulariahodgsonii*), emerged on 19-VIII-2023 (Fig. 17N); • 5 adults, Hiruzen, Maniwa, Okayama Pref., 30-VII-2018 (as larva on *Ligulariafischeri*), emerged on 2–30-VII-2018; • 1 adult, Ashiu, Nantan, Kyoto Pref., 7-VIII-1991 (as larva on *Cirsiumashiuense*), emerged on 20-VIII-1991; 8 adults, Daigonji-kôgen, Matsunoyama, Tôkamachi, Niigata Pref., 19-VIII-2008 (as larva on *Cirsiuminundatum*), emerged on 21–29-VIII-2008; • 5 adults, Aburazaka, Ôno, Fukui Pref., 6-IX-2019 (as larva on Cirsiumtashiroivar.hidaense), emerged on 4–7-X-2019; • 5 adults, Koigakubo, Tessai, Niimi, Okayama Pref., 9-X-2017 (as larva on Cirsiumtashiroivar.hidaense), emerged on 17-X-2017; • 5 adults, Niken-chaya, Shizuichi, Sakyô, Kyoto Pref., 10-VI-2015 (as larva on *Cirsiumkiotoense*), emerged on 30-VI-2015; 1 adult, Mt. Hakusan, Shiramine, Hakusan, Ishikawa Pref., 27-VII-1998 (as larva on *Cirsiummatsumurae*), emerged on 20-VIII-1998; • 1 adult, Kaida-kôgen, Kiso, Nagano Pref., 7-VIII-2016 (as larva on *Cirsiumoligophyllum*), emerged on 30-VIII-2016; • 1 adult, Toyohara, Nasu, Tochigi Pref., 30-VII-2018 (as larva on *Cirsiummakinoi*), emerged on 3–20-VIII-2018; • 2 adults, Doai, Minakami, Tone-gun, Gunma Pref., 30-VII-2018 (as larva on *Cirsiummicrospicatum*), emerged on 3–20-VIII-2018; • 16 adults, Bibi, Chitose, Hokkaidô, 30-VI-2023 (as larva on *Petasitesjaponicus*), emerged on 24–26-VII-2023; 20 adults, Beppo, Nemuro, Hokkaidô, 23-VII-2018 (as larva on *Petasitesjaponicus*), emerged on 2–27-VIII-2018; • 6 adults, Kisofukushima, Kio Nagano Pref., 19-VII-1999 (as larva on *Petasitesjaponicus*), emerged on 2–11-VIII-1999;

#### ﻿ Dactylispa angulosa

Taxon classificationAnimaliaColeopteraChrysomelidae

(Solsky, 1872)

1578AC17-D146-50A3-AF89-DBF1AA12F4EB

Fig. 17O–X

##### Host plant.

Lamiaceae: Clinopodiumchinense(Benth.)var.parviflorum (Kudo), *C.gracile* (Benth.), C.micranthum(Regel)var.sachalinense (F.Schmidt), *Isodoninflexa* Kudo, *Glechomahederacea* (A.Gray), *Prunellavulgaris* L., *Salviaglabrescens* (Franch. et Sav.), *S.japonica* Thunb., *S.ranzaniana* Makino; Boraginaceae: *Lithospermumzollingeri* (A. DC.).

##### Leaf mine.

Upper-layer aggregate blotch mine on mature leaf (Fig. 17Q, R, U, V). Frass is granular, deposited throughout the mine. The fully grown larva pupates in the mine.

##### Material examined.

Lamiaceae: • 2 adults, Sabushi-gawa, Niimi, Okayama Pref., 20-VI-2020 (as larva on *Salviaranzaniana*), emerged on 19–24-VII-2020 (Fig. 17O–R); • 4 adults, Toyohara, Nasu, Tochigi Pref., 2-VII-2022 (as larva on *Salviaglabrescens*), emerged on 19–25-VII-2022 (Fig. 17S–U); • 1 adult, Omotsubo, Tetta, Niimi, Okayama Pref., 19-VII-1991 (as larva on *Salviajaponica*), emerged on 21-VII-1991; • 3 adult, Kaida-kôgen, Kiso, Nagano Pref., 3-VIII-2002 (as larva on *Prunellavulgaris*), emerged on 21–28-VIII-2002; • 9 adults, Chigonosawa, Kisofukushima, Kiso, Nagano Pref., 20-VII-1997 (as larva on Clinopodiumchinensevar.parviflorum), emerged on 28-VII–1-VIII-1997; • 1 adult, Aburazaka, Ôno, Fukui Pref., 11-VII-2003 (as larva on Clinopodiummicranthumvar.sachalinense), emerged on 1-VIII-2003; • 1 adult, Nagawado, Matsumoto, Nagano Pref., 10-VIII-2016 (as larva on *Clinopodiummicranthum*), emerged on 27-VIII-2016 (Fig. 17V); 1 adult, Chigonosawa, Kisofukushima, Kiso, Nagano Pref., 20-VII-1995 (as larva on *Isodoninflexus*), emerged on 5-VIII-2016; • 1 adult, Chigonosawa, Kisofukushima, Kiso, Nagano Pref., 20-VII-1995 (as larva on *Glechomahederacea*), emerged on 10-VIII-1995; • 1 adult, Mumyôdani, Tetta, Niimi, Okayama Pref., 9-VII-1991 (as larva on *Meehaniaurticifolia*), emerged on ?-VIII-1991.

Boraginaceae: • 1 adult, Taishaku-kyô, Shôbara, Hiroshima Pref., 8-VII-1991 (as larva on *Lithospermumzollingeri*), emerged on ?-VIII-1991 (Fig. 17W–X).

#### ﻿ Dactylispa higoniae

Taxon classificationAnimaliaColeopteraChrysomelidae

(Lewis, 1896)

CA68E110-3279-5747-91EA-37A251D65E30

Fig. 18A–H

##### Host plant.

Boraginaceae: *Callicarpamollis* Siebold et Zucc.

##### Leaf mine.

**Figure 18.**
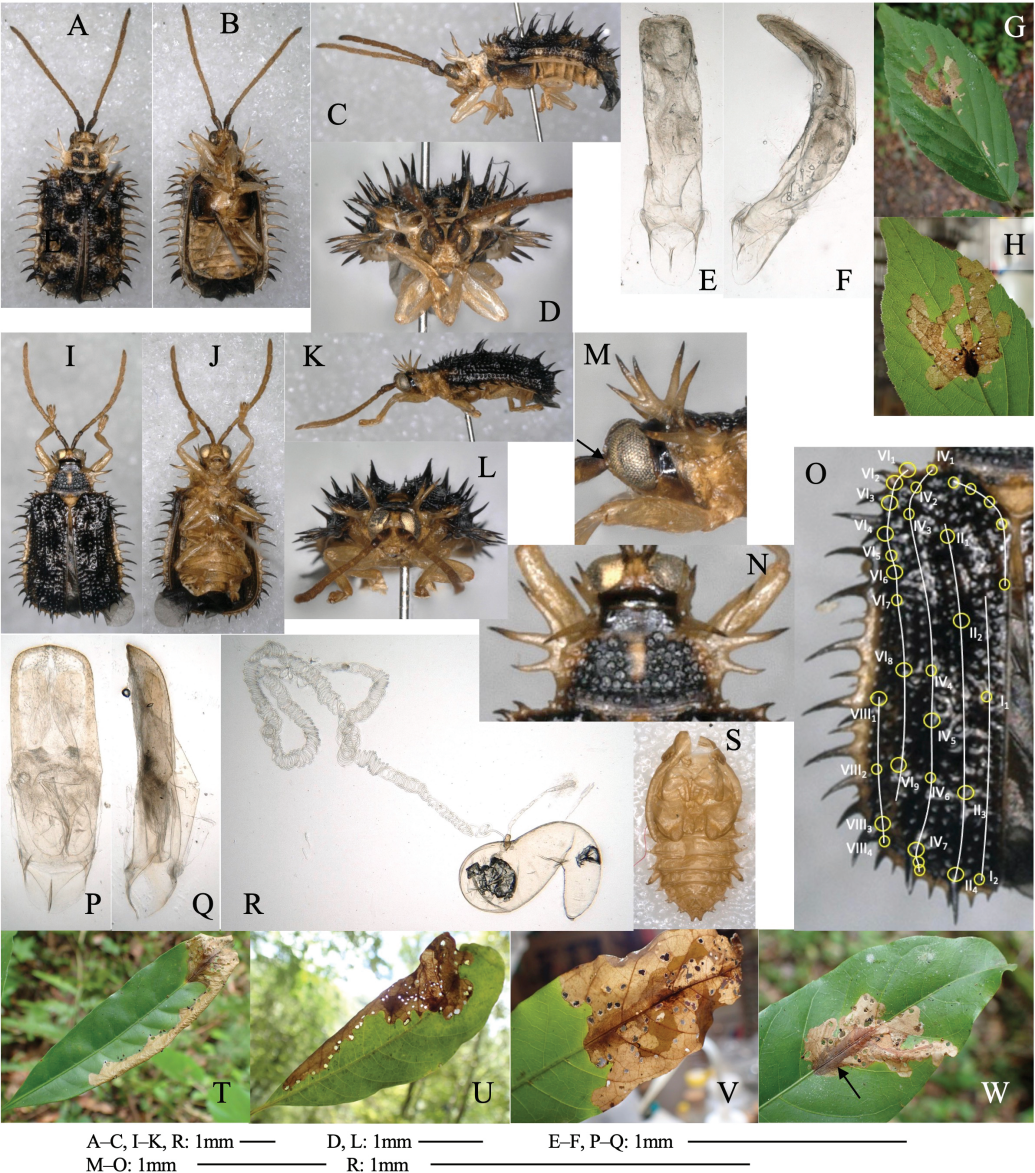
Adult morphology, male genitalia, and leaf mines of two *Dactylispa* species **A–H***D.higoniae***I–W***D.adinae* sp. nov. **A–L** dorsal (**A, I**), ventral (**B, J**), lateral (**C, K**), frontal (**D, L**) views of adults **M** lateral view of prothorax **N** dorsal view of prothorax **O** arrangement of spines on interstices I–VIII of left elytra (yellow circles denote spines on interstices, size of circle indicates length of spine) **E, F, P–Q** aedeagus in male genitalia (**E, P** ventral, **F, Q** lateral) **R** spermatheca and ductus spermatheca in female genitalia **S** ventral view of pupal exuvia **G, H, T–W** leaf mines. Host plants **G, H***Callicarpamollis***T–W***Adinapilulifera* at Inohae, Nichinan, Miyazaki Pref. An arrow in **W** indicates pupation chamber.

Upper-layer radiate mine on mature leaf, usually mining at the central part of the leaf (Fig. 18G, H). The mine has many holes perforated by larva along the margin of the mine, and frass is excreted outside through the holes. The fully grown larva pupates at central area just on midrib in the mine.

##### Material examined.

Lamiaceae: • 2 adults, Inohae, Nichinan, Miyazaki Pref., 24-VII-2021 (as larva on *Callicarpamollis*), emerged on 31-VII–1-VIII-2021 (Fig. 18A–H).

#### ﻿ Dactylispa adinae

Taxon classificationAnimaliaColeopteraChrysomelidae

Kato sp. nov.

C7C0D40A-3420-5B64-AD54-F122E904E9B0

https://zoobank.org/299A7AE8-FE0C-4B87-8B81-133D498C38D1

Fig. 18I–W

##### Type locality.

Japan: Miyazaki Pref., Nichinan, Inohae.

##### Type material.

***Holotype***: • ♂, Inohae, Nichinan, Miyazaki Pref. (31.728°N, 131.369°E, 85 m above sea level), 18-VII-2018 (as larva on *Adinapilulifera* collected by M. Kato), emerged on 1-VIII-2018 (NSMT-I-C-200360). ***Paratypes***: • 1 ♂ 2 ♀ same as holotype, emerged on 31-VII–6-VIII-2018 (NSMT-I-C-200361–200363); 1 ♀ Kaeda-keikoku, Miyazaki, Miyazaki Pref., 11-IV-2021 (as larva on *Adinapilulifera* by M. Kato), emerged on 26-V-2021 (NSMT-I-C-200365).

##### Additional material examined.

• 1 ♂ 1 ♀ same as holotype, emerged on 3–14-VIII-2021; • 1 ♂ Kaeda-keikoku, Miyazaki, Miyazaki Pref., 11-IV-2021 (as larva on *Adinapilulifera*), emerged on 31-V-2021.

##### Diagnosis.

This newly identified species exhibits a rectangular, flattened morphology (length: 4.6–4.7 mm), characterized by black, spiny pronotum and elytra, as well as a dull yellow ventral surface. The pronotum bears a pair of dull yellow trifurcate anterior spines and three pairs of dull yellow long spines oriented horizontally. Similarly, the elytron exhibits numerous long and short spines along margins and interstices I, II, IV, VI, and VIII. The male genitalia feature an aedeagus resembling a spatula in dorsal view, appearing almost parallel-sided and uncurved in lateral view. The larvae mine the leaves of Rubiaceae, particularly *Adinapilulifera*. This species resembles *Dactylispanigrodiscalis* Gressitt, 1938, a Chinese species. However, it is differentiated from the latter by the widely separated anterior trifurcate spines of the prothorax and the basally separated first and second lateral spines on the prothorax.

##### Description.

**Male** (Fig. 18I–Q). ***Habitus*.** The body is 4.6–4.7 mm in length (excluding spines) and is mostly black on the dorsal surface and dull yellow on the ventral surface and head (Fig. 18I, J). The pronotum is black except for dull yellow margins, spines, and a medial linear area before the posterior margin. The elytra are largely black except for dull yellow margins in the middle.

***Head*.** The head is broader than the anterior margin of the prothorax, narrowing behind the eyes and with a black, smooth, and shining occiput. The frons is yellowish-brown and rugose, with a small projection between antennal insertions (Fig. 18M, arrow). The antennae are moderately long, approximately three-quarters of the body length. The segments do not feature spines and are covered with fine short hairs. Segment 1 is the longest among the 11 segments and is slightly curved outward. Segments 1 and 2 are dark brown, whereas segments 3–11 are yellowish brown. Segments 5–11 are slightly thicker than segments 3–4 (Fig. 18I). The proportional lengths of antennomeres 1–11 are as follows: 1:0.3:0.5:0.5:0.6:0.6:0.6:0.6:0.6:0.6:0.9.

***Thorax*.** The pronotum is transverse, measuring 1.6-fold as wide as long, with rounded sides that are prominently produced and flattened medially (Fig. 18I). A pair of dull yellow trifurcate spines appears at the anterior margin of the pronotum (Fig. 18K, M). The two anterior projections share a long common stem and point upward, whereas the posterior projection points diagonally upward and backward, with black apexes. The lateral margin of the pronotum features three long, dull yellow spines pointed horizontally, with the anterior two being longer than the posterior one and located basally on a common stem. The apexes of the anterior two projections are black (Fig. 18N). The base of the disc features a transverse impression. The disc is granulated and covered with large punctures between the lateral spines, with a dull yellow, impunctate, longitudinal linear depression before the posterior margin along the median line.

The scutellum is finely granulose and broad but narrow and subangulate posteriorly. The elytra are largely parallel-sided and broadly rounded posteriorly, with distinct rounded punctures on the surface (Fig. 18I) that are largely black. However, the lateral margins in the middle and apical regions (including spines) are dull yellow. The base of the elytron is wider than the pronotum, with the sides and disc bearing numerous long spines. The lateral margin of the elytron is flattened on each side, featuring 15 or 16 compressed spines, with alternating long and short spines. Each long spine is as long as the first segment of the antenna and slightly curved backward. The apical margin is covered with seven or eight short spines. The elytral interstices are covered with long and short spines (Fig. 18K, O): interspace I with two short spines, interspace II with four long spines, interspace IV with nine spines of which the fifth and seventh are long, interspace VI with nine spines of which the fifth and seventh are short, and interspace VIII with four spines of which the first and third are long. Punctuation is regular and coarse, with the distance between punctures being smaller than the puncture diameter. The legs are dull yellow and slender.

***Abdomen*.** The abdomen is dull yellow.

***Genitalia*.** The aedeagus has a spatula-like appearance in dorsal view, with a poorly sclerotized basal region (Fig. 18P, Q). It measures 3-fold longer than its width, appearing almost parallel-sided and narrowed to a rounded apex. It is almost uncurved in lateral view. There are V-shaped phallobase apodeme rings around and keeling the median lobe.

**Female.** The body of females is larger than that of males, measuring 4.7–5.1 mm in length.

***Genitalia*.** The spermatheca is J-shaped and swollen (Fig. 18R). The cornu is gradually narrowed toward the blunt apex. The ductus spermatheca is thin, exceedingly elongated, and regularly and tightly coiled.

***Pupa*.** The body is pale brown, elongated, and flattened dorsoventrally (Fig. 18S). Abdominal segments I–IV feature acuminate bifurcated lateral processes at the apical region, with the ventral process smaller than the dorsal process. The processes of segment IV are significantly enlarged, and the ventral process is particularly thick and projecting diagonally backward, with a hooked tip. Segments V–VII feature bifurcated processes, with greater bifurcation of the ventral processes. Segments VIII and IX are fused, exhibiting two blunt processes apically.

##### Distribution.

Around Obi, Southern Miyazaki Prefecture, Japan.

##### Host plants.

Rubiaceae: *Adinapilulifera* (Lam.).

##### Leaf mine.

Full-depth, linear-blotch mine on mature leaf, often transitioning into a radiate mine (Fig. 18T–W). The larvae create holes that penetrate the upper and lower layers of the leaf, with frass being discharged outside through the holes. Holes form intermittent lines along the sides of the mine. Larvae sometimes exit the mine and move to another leaf to construct a new mine. Fully grown larvae pupate in the pupation chamber located nearly at the center of the mine, typically on the midrib. The upper layer of the leaf around the chamber remains undamaged and green.

##### Etymology.

The species name refers to the genus name of the host plant, *Adina*.

##### Japanese name.

Obi-togehamushi.

##### Remarks.

*Dactylispa* exhibits considerable diversity in China (Chen et al. 1961; Chen and Tan 1964). This newly discovered species resembles *Dactylispanigrodiscalis*, a Chinese species, in terms of its habitus, presence of trifurcate anterior spines on the prothorax, arrangement of spines on the elytra (Gressitt 1938), and association with the host plant family (Rubiaceae). However, it is differentiated from the latter by the presence of a pair of anterior trifurcate spines of the prothorax that are widely separated from each other, with the distance between spines being subequal to the width of the occiput but less than three-quarters of the width in the latter. The first and second lateral spines on the prothorax of the newly identified species branch basally, whereas in *D.nigrodiscalis*, the first and second lateral spines on the prothorax share common stems. Moreover, the former is associated with *Adinapilulifera* as the host plant species, whereas the latter is associated with *Metadinatrichotoma*, *Mussaendapubescens*, and *Uncariarhynchophylla* (Yang et al. 2023). Given that *D.nigrodiscalis* belongs to the subgenus Triplispa (Zhang et al. 2021), this new species is considered a member of *Triplispa*.

This newly discovered species resembles *Dactylispaissikii* Chûjô, 1938 in terms of the presence of trifurcate anterior spines on the prothorax. However, it is distinguished from the latter by the presence of yellow spines on the prothorax compared to black in the latter, the presence of a yellow area on the pronotum and elytron compared to a black pronotum and elytron in the latter, completely yellow sternum compared to a dark brown sternum in the latter, more and shorter lateral spines on the elytron, and association with the host plant family Rubiaceae compared to Poaceae in the latter.

Although the new species is similar to *Dactylispahigoniae* in terms of habitus, it is distinguished from the latter by the presence of trifurcate anterior spines on the prothorax compared to bifurcate spines in the latter, yellow propleuron and sternum compared to their dark brown color in the latter except along the longitudinal suture, and association with the host plant family Rubiaceae compared to Lamiaceae for the latter.

#### ﻿ Dactylispa issikii

Taxon classificationAnimaliaColeopteraChrysomelidae

Chûjô, 1938

5EAA5601-2199-5B82-AC89-A7E0F92C760F

Fig. 19A–H

##### Host plant.

Poaceae: Pleioblastuschinovar.viridis (Makino), *P.simonii* (Carrière).

##### Leaf mine.

**Figure 19.**
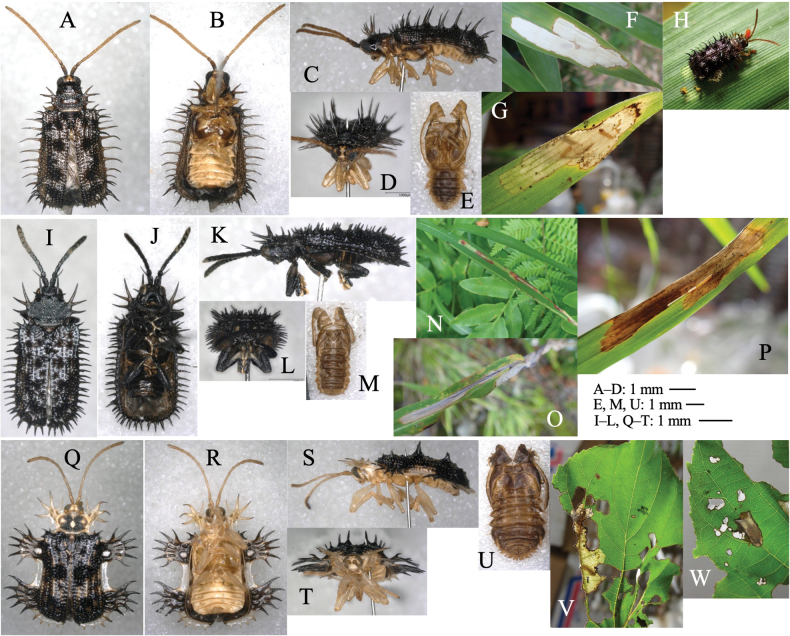
Adult morphology and leaf mines of three Hispini species **A–H***Dactylispaissikii***I–P***Rhadinosanigrocyanea***Q–W***Platypriamelli***A, I, Q** dorsal **B, J, R** ventral **C, K, S** lateral **D, L, T** frontal views of adults **E, M, U** exuviae **F, G, N–P, V, W** leaf mines. Host plants **G, H***Pleioblastuschino* at Tsuge, Kuma, Kumamoto Pref. **B–O***Miscanthustinctorius* at Hatabe, Kumamoto Pref. **P***Miscanthustinctorius* at Fukube-dani, Hakusan, Ishikawa Pref. **V, W***Hoveniadulcis* at Kin, Tsushima Is., Nagasaki Pref.

White full-depth linear-blotch mine on mature leaf, usually situated around the leaf tip (Fig. 19F, G). Frass is granular, deposited linearly along either side of the mine, or discharged outside from a slit made along mine margin. The mine contains one or a few larvae, and the fully grown larva pupates in the mine.

##### Material examined.

• 8 adults, Tsuge, Ashikita, Ashikita-gun, Kumamoto Pref., 16-VII-2018 (as larva on Pleioblastuschinovar.viridis), emerged on 20-VII–6-VIII-2018 (Fig. 19A–H); • 1 adult, Kyoto University, Yoshida, Sakyo, Kyoto Pref., 11-X-2015 (as larva on P.chinovar.viridis), emerged on 15-XI-2015; • 6 adults, Fushimi-inari, Fushimi-ku, Kyoto Pref., 12-VII-2019 (as larva on P.chinovar.viridis), emerged on 16–30-VII-2019; • 5 adults, Inohae, Nichinan, Miyazaki Pref., 14-VII-2021 (as larva on *P.simonii*), emerged on 19-VII–8-VIII-2021;

### ﻿*Hispellinus* Weise, 1897

#### ﻿ Hispellinus moerens

Taxon classificationAnimaliaColeopteraChrysomelidae

(Baly, 1874)

D53BC85E-5D51-569F-A6CD-1E283423C068

##### Host plant.

Poaceae: *Miscanthussinensis* Anderss. (Hayashi et al. 1984; Kimoto and Takizawa 1993).

##### Leaf mine.

Unknown.

### ﻿*Rhadinosa* Weise, 1905

#### ﻿ Rhadinosa nigrocyanea

Taxon classificationAnimaliaColeopteraChrysomelidae

(Motschulsky, 1860)

9A15B03B-5D6D-55ED-8A79-25D0A42B0CE5

Fig. 19I–P

##### Host plant.

Poaceae: *Miscanthuscondensatus* Hack., *M.sinensis*, *M.oligostachyus* Stapf, *M.tinctorius* (Steud.), Pleioblastuschinovar.viridis.

##### Leaf mine.

Upper-layer linear-blotch mine on mature leaf (Fig. 19N–P). Frass is granular, deposited along larval trajectory in the mine. The fully grown larva pupates in the mine.

##### Material examined.

• 1 adult, Hatabe-gen’ya, Aso, Kumamoto Pref., 16-VII-2018 (as larva on *Miscanthussinensis*), emerged on 21-VII-2018 (Fig. 19I–O); • 1 adult, Fukube-dani, Hakusan, Ishikawa Pref., 1-X-2019 (as larva on *M.tinctorius*), emerged on 14-X-2019 (Fig. 19P); • 2 adults, Mt. Kujû, Kokonoe, Kusu-gun, Ôita Pref., 27-IX-2019 (as larva on *M.oligostachyus*), emerged on 7–11-X-2019; • 1 adult, Mt. Yufu, Beppu, Ôita Pref., 23-VII-2017 (as larva on Pleioblastuschinovar.viridis), emerged on 26-IX-2017.

### ﻿*Platypria* Guérin-Méneville, 1840

#### ﻿ Platypria melli

Taxon classificationAnimaliaColeopteraChrysomelidae

Uhmann, 1955

6D753692-D1AC-5482-99C2-319D014B8B4B

Fig. 19Q–W

##### Host plant.

Rhamnaceae: *Hoveniadulcis* Thunb.

##### Leaf mine.

Full-depth blotch mine on the mature leaf (Fig. 19V). Frass is granular deposited in the mine. The larva sometimes relocates its mine. The fully grown larva pupates in the mine, sometimes in a mine newly constructed for pupation (Fig. 19W).

##### Material examined.

• 1 adult, Kin, Kamitsushima Is., Tsushima, Nagasaki Pref., 3-VII-2015 (as larva on *Hoveniadulcis* collected by T. Kato), emerged on 9–16-VIII-2015 (Fig. 19Q–W).

### ﻿*Dicladispa* Gestro, 1897

#### ﻿ Dicladispa boutani

Taxon classificationAnimaliaColeopteraChrysomelidae

(Weise, 1905)

7845BEF8-7B14-5F3D-B21D-A55B9966B13E

##### Host plant.

Poaceae: *Oryzasativa* (Kimoto and Takizawa 1987).

##### Leaf mine.

Unknown.

### ﻿Notosacanthini Gressitt, 1952

***Notosacantha* Chevrolat, 1837**

#### ﻿ Notosacantha ihai

Taxon classificationAnimaliaColeopteraChrysomelidae

Chûjô, 1958

AE67ED2B-9289-5B4C-BA64-EC2901FC6073

Fig. 20A–Y

##### Host plant.

Proteaceae: *Heliciacochinchinensis* Lour.; Pentaphylacaceae: *Adinandraryukyuensis* Masam., *Euryajaponica* Thunb.; Theaceae: *Schimaliukiuensis* Nakai; Staphyleaceae: *Turpiniaternata* Nakai; Melastomataceae: *Brediaokinawensis* (Matsum.), *B.yaeyamensis* (Matsum.); Symplocaceae: *Symplocosglauca* (Thunb.), *S.sonoharae* Koidz.; Loganiaceae: *Gardnerialiukiuensis* Hatus. All these plants are woody plants having coriaceous evergreen leaves.

##### Leaf mine.

**Figure 20.**
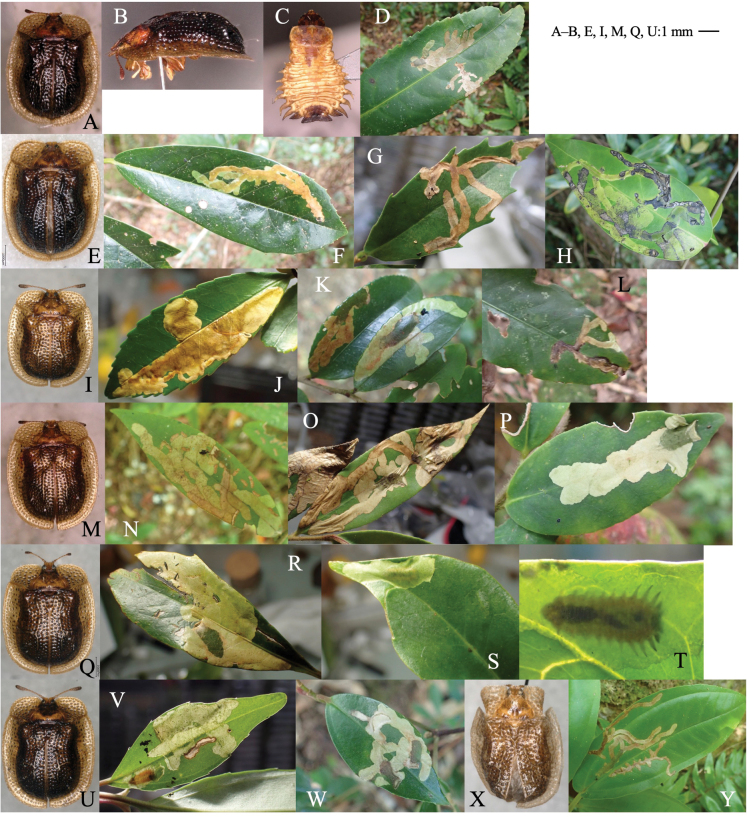
Adult habitus and leaf mines of *Notosacanthaihai* on various host plants **A–D***Turpiniaternata* at Mt. Yuwan, Amami-ôshima, Kagoshima Pref. **E, F***Symplocosglauca* at Yona, Kunigami, Okinawa Pref. **G***Symplocossonoharae* at Mt. Yuwan, Amami-ôshima, Kagoshima Pref. **H***Gardnerianutans* at Mt. Nishime, Kunigami, Okinawa Pref. **I, J***Euryajaponica* at Mt. Nishime, Kunigami, Okinawa Pref. **K***Euryajaponica* at Yona, Kunigami, Okinawa Pref. **L***Schimawallichii* at Mt. Yuwan, Amami-ôhsima, Kagoshima Pref. **M–P***Adinandraryukyuensis* at Yona, Kunigami, Okinawa Pref. **Q–T***Heliciacochinchinensis* at Yona, Kunigami, Okinawa Pref. **U–W***Brediaokinawensis* at Mt. Nishime, Kunigami, Okinawa **X, Y***Brediayaeyamensis* at Komi, Iriomote Is., Okinawa Pref.

Upper-layer linear-blotch, often radiate mine on mature leaf (Fig. 20D, F–H, J–L, N–P, R–T, V–W, Y). The mine often has blind ends and branches, and the outline of the mine is often undulated. The larva sometimes relocates its mine. Frass is granular, often discharged outside from slit made by the larva. The fully grown larva pupates in the mine, especially in the mine newly constructed for pupation.

##### Material examined.

• 1 adult, Mt. Yuwan, Uken, Amami-ôshima Is., Kagoshima Pref., 25-V-2017 (as larva on *Turpiniaternata*), emerged on 15–18-VI-2017 (Fig. 20A–D); • 1 adult, Yona, Kunigami, Okinawa Is., Okinawa Pref., 19-V-2011 (as larva on *Symplocosglauca*), emerged on 13-VI-2011 (Fig. 20E, F); • 2 adults, Mt. Yuwan, Uken, Amami-ôshima Is., Kagoshima Pref., 29-IV-2005 (as larva on *Symplocossonoharae*), emerged on 25-V-2005 (Fig. 20G); • 2 leaf mines, Mt. Nishime, Kunigami, Okinawa Pref., 17-III-2020 on *Gardnerialiukiuensis* (Fig. 20H); • 1 adult, Mt. Nishime, Kunigami, Okinawa Is., Okinawa Pref., 17-III-2020 (as larva on Euryajaponicavar.japonica), emerged on 9-V-2020 (Fig. 20I–K); • 2 leaf mines, Mt. Yuwan, Uken, Amami-ôshima Is., Kagoshima Pref., 23-XI-2023 on *Schimaliukiuensis* (Fig. 20L); • 2 adults, Yona, Kunigami, Okinawa Is., Okinawa Pref., 29-III-2018 (as larva on *Adinandraryukyuensis*), emerged on 4–9-V-2018 (Fig. 20M–P); • 3 adults, Yona, Kunigami, Okinawa Is., Okinawa Pref., 28-III-2018 (as larva on *Heliciacochinchinensis*), emerged on 30-IV–5-V-2018 (Fig. 20Q–T); • 1 adult, Mt. Nishime, Kunigami, Okinawa Is., Okinawa Pref., 17-III-2020 (as larva on *Brediaokinawensis*), emerged on 1-V-2020 (Fig. 20U–W); • 1 adult, Komi, Iriomote Is., Yaeyama, Okinawa Pref., 28-III-2018 (as larva on *Adinandraryukyuensis*), emerged on 4-IV-2018 (Fig. 20X, Y).

#### ﻿ Notosacantha loochooana

Taxon classificationAnimaliaColeopteraChrysomelidae

Chûjô, 1961

55255663-A5AC-558E-89B7-3AB096CC8A56

Fig. 21A–F

##### Host plant.

Iteaceae: *Iteaoldhamii* Schneider; Rubiaceae: *Gardeniajasminoides* Ellis.

##### Leaf mine.

**Figure 21.**
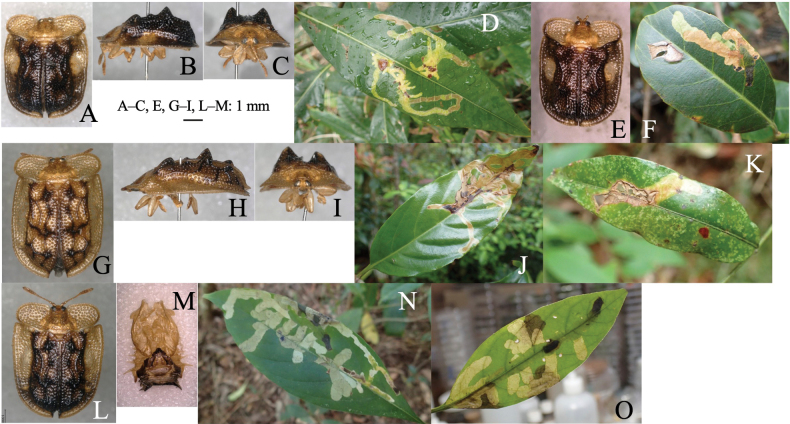
Habitus and leaf mines of two *Notosacantha* species **A–F***Notosacanthaloochooana***G–O***N.nishiyamai***A, G, L** dorsal **B, H** lateral **C, I** frontal view of adults **M** exuvia **D, F, J, K, N, O** leaf mines. Host plants **A–D***Gardeniajasminoides* at Nagakumo-toge, Amami-ôshima, Kagoshima Pref. **E, F***Iteaoldhamii* at Higashinakama, Amami-ôshima Is., Kagoshima Pref. **G–J***Tarennagracilipes* at Nekumachiji, Ôgimi, Okinawa Pref. **K***Coptosapeltadiffusa* at Yona, Kunigami, Okinawa Pref. **L–O***Randiacanthioides* at Yona, Kunigami, Okinawa Pref.

The upper-layer linear-blotch, often radiate mine occurs on the mature leaf (Fig. 21D, F). The mine often has blind ends and branches. The larva sometimes relocates its mine. Frass is granular, often discharged outside from slit made by the larva. The fully grown larva pupates in the mine, often in a mine newly constructed for pupation.

##### Material examined.

• 9 adults, Nagakumo-tôge, Amami-ôshima Is., Kagoshima Pref., 22-II-2015 (as larva on *Gardeniajasminoides*), emerged on 30-IV–1-V-2015 (Fig. 21A–D); 2 adults, Mt. Yui, Setouchi, Amami-ôshima Is., Kagoshima Pref., 19-III-1997 (as larva on *Gardeniajasminoides*), emerged on 1-V-1997; • 1 adult, Higashinakama, Sumiyo, Amami-ôshima, Kagoshima Pref., 21-II-2015 (as larva on *Iteaoldhamii*), emerged on 4-V-2015 (Fig. 21E, F).

#### ﻿ Notosacantha nishiyamai

Taxon classificationAnimaliaColeopteraChrysomelidae

Komiya, 2002

D62CE543-7F55-5EAC-A2A6-DA5944D53884

Fig. 21G–O

##### Host plant.

Rubiaceae: *Coptosapeltadiffusa* (Champ. ex Benth.), *Gardeniajasminoides*, *Randiacanthioides* Champ. ex Benth, *Tarennagracilipes* Ohwi.

##### Leaf mine.

Upper-layer linear-blotch, often radiate mine on mature leaf (Fig. 21J–K, N–O). The mine often has blind ends and branches. The larva sometimes relocates its mine. Frass is granular, often discharged outside from slit made by the larva. The fully grown larva pupates in a pupation site in the mine along the midrib, where upper layer of the leaf is kept intact and green (Fig. 21O).

##### Material examined.

• 1 adult, Mt. Nekumachiji, Ôgimi, Okinawa Is. Okinawa Pref., 30-III-2018 (as larva on *Tarennagracilipes*), emerged on 2-V-2018 (Fig. 21G–J); • 1 adult, Mt. Nishime, Kunigami, Okinawa Is., Okinawa Pref., 22-XII-1989 (as larva on *Gardeniajasminoides*), emerged on ?-IV-1990; • 2 leaf mines, Yona, Kunigami, Okinawa Is., Okinawa Pref., 29-III-2019 on *Coptosapeltadiffusa* (Fig. 21K); • 2 adults, Yona, Kunigami, Okinawa Is., Okinawa Pref., 29-III-2018 (as larva on *Randiacanthioides*), emerged on 3-V-2018 (Fig. 21L–O).

## ﻿Discussion

### ﻿Pattern of host plant utilization

Our study reviews the 64 leaf-mining beetle species of Chrysomeloidea within the Japanese Archipelago (Table 1), including the first description of leaf mines for 30 species. These species exhibit varied associations, including larvae found on pteridophytes (one species on Equisetales and four on Polypodiales), cycads (two species on Cycadales), and diverse angiosperms (66 species with 24 orders of angiosperms) (Table 2). Host plants are documented for the first time for six species: *Longitarsusholsaticus*, *Diboliajaponica*, *Psylliodessubrugosa*, *Halticorcusduodecimmaculata*, *Sphaerodermakomiana*, and *Dactylispaadinae* (Table 1). Host plant records are analyzed with the Chrysomelidae phylogeny of Nie et al. (2020), and suggest that host shifts from angiosperms to pteridophytes have likely occurred once or twice, in *Hippuriphila* and *Halticorcus* (Table 2). The mined part of *Equisetum* by *Hippuriphila* is a functional leaf but a botanical shoot. The larva of *Hippuriphila* do not exhibit the typical larval body flattering characteristic observed in most leaf miners. Similar non-flattened leaf-mining larvae are found in the cerambycid larvae mining megasporophyll and leaf stalk of cycads.

**Table 2.** Host plant orders associated with Japanese leaf-mining species of Chrysomeloidea. Plant orders are arranged according to the system described by Ruggiero et al. (2015).

Beetle family	Genus	Species	Plant order																										
			Equisetales	Polypodiales	Cycadales	Piperales	Dioscorales	Pandanales	Liliales	Poales	Commelinales	Ranumculales	Proteales	Saxifragales	Celastrales	Oxalidales	Malpighiales	Rosales	Fagales	Myrtales	Crossosomatales	Brassicales	Caryophyllales	Ericales	Gentianales	Boraginales	Lamiales	Asterales	Total no. orders
Cerambycidae	*Mimectatina*	*meridianaohirai*			1																								1
	*Sybra*	*ordinata*			1																								1
Megalopodinae	*Zeugophora*	*annulata*													1														1
		*chujoi*													1														1
		*flavonotata*													1														1
		*nigricolis*													1														1
		*unifasciata*													1														1
		*varipes*																						1					1
		*hozumii*															1												1
		*japonica*															1												1
		*cupka*															1												1
Chrysomelidae	*Phyllotreta*	*ezoensis*																				1							1
		*shirahatai*																				1							1
	*Longitarsus*	*holsaticus*																				1							1
	*Dibolia*	*japonica*																									1		1
	*Mantura*	*clavareaui*																					1						1
		*fulvipes*														1													1
	*Hippuriphila*	*babai*	1																										1
	*Psylliodes*	*chujoe*																				1							1
		*punctifrons*																				1							1
	*Halticorcus*	*kasuga*		1																									1
		*sauteri*		1																									1
		*hiranoi*		1																									1
		*duodecimmaculata*		1																									1
	*Argopistes*	*coccinelliformis*																									1		1
		*biplagiata*																									1		1
		*unicolor*																									1		1
		*ryukyuensis*																									1		1
		*tsekooni*																									1		1
	*Argopus*	*balyi*										1																	1
		*clypeatus*										1																	1
		punctipennis*				1						1																1	3
	*Sphaeroderma*	nigricolle*					1	1	1																				3
		*japanum*									1																		1
		*tarsatum*								1																			1
		*seriatum*								1																			1
		*apicale*								1																			1
		*akebia*										1																	1
		*inaizumii*										1																	1
		*placidum*										1																	1
		*unicolor*										1																	1
		*uenoi*										1																	1
		*separatum*										1																	1
		*quadrimaculatum*										1																	1
		*flavonotatum*										1																	1
		*ohkuboi*										1																	1
		*balyi*																										1	1
		*komiana* sp. nov.																										1	1
	*Liptispa*	*taguchii*								1																			1
		*miyamotoi*								1																			1
	*Asamangulia*	*yonakuni*								1																			1
	*Dactylispa*	*subquadrata*																	1										1
		*masonii*																										1	1
		angulosa*																								1	1		2
		*higoniae*																								1			1
		*adinae* sp. nov.																							1				1
		*issikii*								1																			1
	*Hispellinus*	*moerens*								1																			1
	*Rhadinosa*	*nigrocyanea*								1																			1
	*Platypria*	*melli*																1											1
	*Dicladispa*	*boutani*								1																			1
	*Notosacantha*	ihai*											1							1	1			1					4
		loochooana*												1											1				2
		nishiyamai*																							1				1
Total number of beetle species				4	2	1	1	1	1	10	1	12	1	1	5	1	3	1	1	1	1	5	1	2	3	2	7	4	72

* The beetle species exibiting extended host specificity.

All four *Halticorcus* species are associated with epiphytic or terrestrial evergreen ferns characterized by simple or unipinnate compound leaves, most of which are large, coriaceous, and sometimes fleshy. Each species uses multiple fern species, with *H.sauteri* utilizing specimens from six genera belonging to three families. While some *Halticorcus* species share host plant genera and species, sympatric species (e.g., *H.hiranoi* and *H.sauteri*) do not typically share the same host plant species, suggesting that host plant segregation likely played a role in speciation of *Halticorcus*.

Within the beetle clades associated with angiosperms, diversification has been noted in Polypodiales (*Halticorcus*), Rununculaceae (*Argopus* and *Sphaeroderma*), Celastraceae (*Zeugophora*), and Oleaceae in Lamiales (*Argopistes*) (Table 2). This suggests that diversification may have occurred through host shifts among different plant species and genera within the same genus or family. However, within each beetle genus, multiple species can share the same plant species. Rearing records suggest that diversification can also occur through the variations in the larval active seasons on the same host plants, without necessitating host shifts among plant species. For example, among the three *Zeugophora* species (*Z.annulata*, *Z.nigricolis*, and *Z.unifasciata*) associated with *Euonymussieboldianus* (Fig. 2A–D, N–Q), *Z.annulata* exclusively mines newly opened leaves in early spring, *Z.nigricolis* targets fully opened leaves in June, and *Z.unifasciata* mines mature leaves between July and September.

### ﻿Extended host specificity

Among the 64 species for which host plants were identified, 29 were specific to particular host species, 12 to host genera, 16 to host families, two to host orders, and five species were non-specific even to order level (Tables 1, 2). In species associated with multiple plant orders or families, each beetle species utilizes only a small number of plant genera or species, suggesting that they are far from generalists. This pattern of host selection can be referred to as extended host specificity, where species are restricted to host plant genera or species belonging to distinct plant orders and families.

*Argopuspunctipennis* is a large red leaf-beetle commonly found on thistle. Our study revealed that this species is associated with 24 plant species distributed among three phylogenetically isolated genera (*Asarum*, *Aconitum*, and *Cirsium*) belonging to different families (Aristolochiaceae, Ranunculaceae, and Asteraceae) and orders (Piperales, Ranunculales, and Asterales) (Fig. 11A–S), illustrating extended host specificity. The combination of these three plant genera is intriguing, particularly because *Aconitum* contains diverse highly toxic metabolites (Ali et al. 2021) and is rarely infested by herbivores. Given that the other two *Argopus* species are associated with *Clematis* (Ranunculaceae), we hypothesize that an intra-familial host shift from *Clematis* to *Aconitum* occurred initially in *A.punctipennis*. Subsequently, inter-familial host shifts occurred, from *Aconitum* to *Asarum* and *Cirsium*. These beetles reared from various plant genera cannot be distinguished, even in terms of male genital morphology, suggesting that host races have not yet been differentiated.

**Figure 22.**
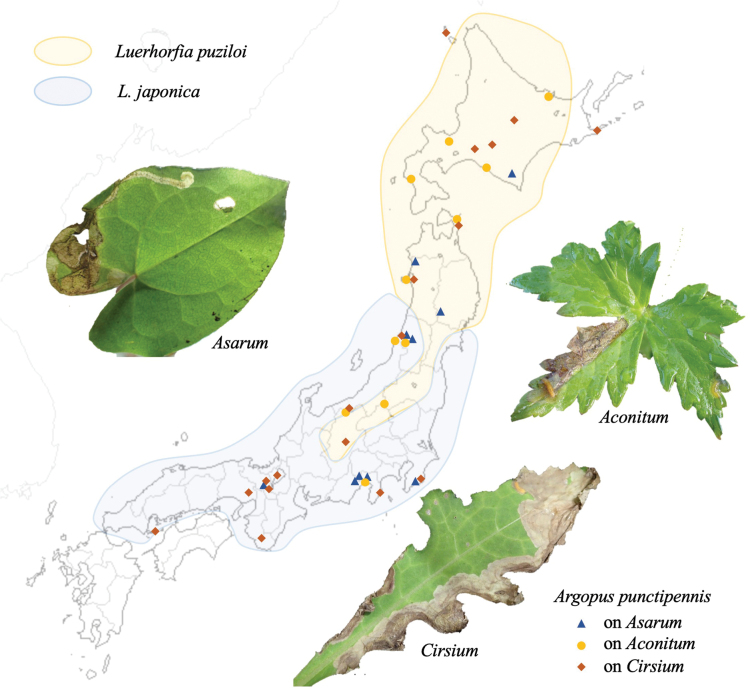
Map showing collection sites of *Argopuspunctipennis* in the Japanese Archipelago with each recorded host plant genus (*Asarum*, *Aconitum* and *Cirsium*) indicated by different symbols. The distributions of two *Asarum*-associated butterfly species, *Luerhdorfiapuziloi* and *L.japonica*, are presented (Suzuki et al. 2023).

The distribution of *Argopuspunctipennis* (Fig. 22) suggests that the two or three plant genera are sympatric and simultaneously utilized at several sites, with no geographical patterns of host utilization. To explain why these three phylogenetically isolated genera are utilized by beetles, chemical analysis of secondary metabolites among host plant genera, bioassays of the beetles against these chemicals, genetic analysis of beetle populations, and phylogenetic analyses of individuals associated with different plant species or genera would be necessary.

In Japan, the host plant genus *Asarum* (Aristolochiaceae) comprises locally restricted diverse species belonging to three sections: deciduous *Asarum* and *Asiasarum* and evergreen *Heterotropa* (Okuyama et al. 2020). Species from the Asiasarum section and several species from Heterotropa are utilized by two butterfly species, *Luerhdorfiapuziloi* Leech, 1889 and *Luerhdorfiajaponica* Leech, 1889 (Papilionidae), which utilize *Asiasarum* and *Heterotropa*, respectively. A recent molecular study revealed that the differential adaptation to various *Asarum* sections between the two *Luehdorfia* species and the reproductive interference between these species have influenced the formation of their current parapatric distribution (Suzuki et al. 2023). In contrast to *Luehdorfia*, *A.punctipennis* utilizes all three groups of *Asarum*, i.e., section Asiasarum in the *L.puziloi* range and sections *Heterotropa* and *Asarum* in the *L.japonica* range (Fig. 22). The difference in geographical host utilization patterns between *Luerhdorfia* and *A.punctipennis* may be attributable to variation in the time spent associating with *Asarum*. The association between *Luerhdorfia* and *Asarum* species is ancient, with divergence occurring approximately 17 million years ago (Suzuki et al. 2023). Conversely, the association between *A.punctipennis* and *Asarum* is of more recent origin, involving a host switch to *Asarum*.

The host genus *Cirsium* (Asteraceae) has undergone significant radiation in the Japanese archipelago (Ohashi et al. 2015), with most of the 150 recorded species being newly endemic. Diverse *Cirsium* species are utilized by *A.punctipennis* from Hokkaidô to Honshû. Additionally, *Cirsium* is utilized by two endemic phytophagous coccinellid beetle species, *Epilachnapustulosa* Kôno, 1937 and *Epilachnaniponica* Lewis, 1896, in Hokkaidô and Honshû, respectively (Katakura, 1997). Recent phylogenetic analyses have revealed that the *Cirsium*-associated *Epilachna* species derived from a Solanaceae-associated clade, which diverged from Cucurbitaceae-associated Asian clades (Katoh et al. 2014). These results suggest that the routes and timing of host shifts to *Cirsium* differ between *Argopus* and *Epilachna*; the host shift in the former occurred more recently from Ranunculaceae, without differentiation between populations on different host plants. Conversely, in the case of *Epilachna*, the host shift from Solanaceae occurred earlier, resulting in the differentiation of *Cirsium*-associated species and subsequent differentiation of the two allopatric species.

**Figure 23.**
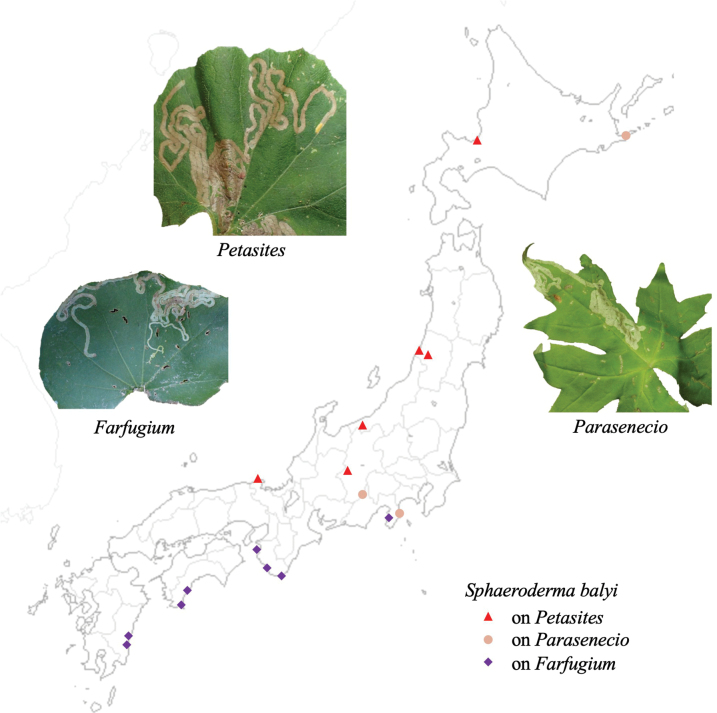
Map showing collection sites of *Sphaerodermabalyi* in the Japanese Archipelago with each recorded host plant genus (*Petasites*, *Parasenecio* and *Farfugium*) indicated by different symbols.

Leaf-mining beetle species often utilize distinct host plant species at different sites within their range of distribution. *Sphaerodermabalyi* utilizes three genera of the tribe Senecioneae within the Asteraceae family, most of which are perennials with large, round leaves (Fig. 16A–J) that are widely distributed in various climatic zones. These host plant genera vary among climatic zones. In cool temperate forests, the beetle species utilize deciduous perennials belonging to *Parasenecio* and *Petasites*, whereas in warm temperate forests, they utilize evergreen perennials belonging to *Farfugium*, with no overlap in their ranges (Fig. 23). Although the leaves of these host plant genera differ in terms of deciduousness, thickness, toughness, shape, and surface coating, their mining patterns are similar, suggesting that the expansion of the host range of *S.balyi* has occurred recently and is ongoing.

In Cassidinae, leaf-mining habit has evolved in two tribes Notosacanthini and Hispini, which respectively comprises 254 and 611 species in the world (Chaboo 2007). In Notosacanthini, extended host specificity was observed for all Japanese species of *Notosacantha* (Cassidinae: Notosacanthini), which construct radiate mines on coriaceous leaves of evergreen woody plants (Figs 20, 21). For example, *N.ihai* utilizes seven restricted genera belonging to seven phylogenetically isolated plant families as host plants. Similar pattern of host association is reported in several tropical *Notosacantha* species (Monteith et al. 2021). Cassidine leaf beetles, including *Notosacantha*, harbor host-specific gammaproteobacterial symbionts in gut-associated symbiotic organs, which help the host to digest food plants by producing pectin-degrading enzymes (Fukumori et al. 2022). In contrast with Notosacanthini, beetles in Hispini do not harbor gammaproteobacterial symbionts and show higher host specificity, suggesting that gammaproteobacterial symbionts may contribute to the extended host specificity of *Notosacantha*.

### ﻿Mining patterns

Characteristic mining patterns described by Frost (1924) as being common to chrysomeloidean leaf miners was not found universally. Instead, their leaf mines are linear, linear-blotch, blotch, or radiate mines (Table 1). Larvae of Megalopodidae and Galerucinae are slightly flattened but still thick, and typically construct full-depth leaf mines, whereas larvae of Cassidinae are sufficiently flattened to construct upper-layer leaf mines. The pupation site is internal in Cerambycidae and Cassidinae but external (i.e., underground) in Megalopodidae and Galerucinae. Frass is typically left behind within its mine in a granular, linear, or meandering linear fashion in most groups except for several species of Hispini in Cassidinae, where frass is discharged outside through perforated holes. Abandoning and reconstructing mines (i.e., mine moving) are observed in various genera in Chrysomelidae, particularly on small leaves and those that wilt easily.

Unique midrib mining behavior was observed in *Zeugophorachujoi*, whose young instar larvae enter the midrib and cause the fall of newly opened leaves of *E.fortunei* (Fig. 2G–I). Similar midrib/petiole mining behaviors in young instars have been observed in a few buprestid species associated with Symplocaceae (Kato and Kawakita 2023). Another petiole/midrib miner is *Psylliodespunctifrons*, whose larvae mine the petiole/midrib of mature leaves of crucifer but do not cause leaf fall or wilting (Fig. 7C–E, H–J, L–P). The larva is not flattened but elongated, and its mine is not sufficiently thick to cause profound leaf damage. This slender larval morphology suggests that the leaf-mining habit has recently evolved from stem or root-mining habits, as *Psylliodes* species associated with the Brassicaceae are internal feeders in stems or roots (Gikonyo et al. 2019). Among the nine *Psylliodes* species in Japan, three (*P.punctifrons*, *P.subrugosa*, and *Psylliodessasakii*) are associated with the Brassicaceae (Takizawa 2005, 2015), and at least two are confirmed to be leaf miners.

Similar to *Psylliodes*, *Phyllotreta* is also associated with Brassicaceae and comprises leaf-mining, stem-boring, and root-boring species (de Jong et al. 2009; Ellis 2020). There are five *Phyllotreta* species in Japan, and their identification is difficult due to the polymorphism of elytral patterns (Takizawa 2007). Our rearing records suggest that at least two *Phyllotreta* species have leaf-mining habits (Fig. 3A–M), and *P.ezoensis* exhibits elytral polymorphism (Fig. 3A, B).

### ﻿Evolution of leaf-mining habits

In Chrysomeloidea, cerambycid and megalopodid larvae are respectively wood borers and leaf-miners, while chrysomelid larvae live external or internal life on plants. Our results revealed that there are at least three leaf-mining clades in Japanese Chrysomelidae: a part of the tribe Alticini in Galerucinae (10 genera), and all members of the tribe Hispini (7 genera) and the tribe Notosacanthini (1 genus) both in Cassidinae (Table 1). Because phylogenetic tree of Chrysomeloidean families is (Chrysomelidae (Megalopodidae, Cerambycidae)) (Cai et al. 2022), and because extant basal clades of Chrysomelidae are external feeders (Nie et al. 2020), leaf-mining life in Chrysomelidae is thought to have evolved from external life in several clades in Alticini and at the base of the two tribes Hispini and Notosacanthini. Further phylogenetic study on Alticini will reveal history of evolutionary shifts between internal and external life in Chrysomelidae.

## Supplementary Material

XML Treatment for Mimectatina meridiana ohirai

XML Treatment for Sybra ordinata

XML Treatment for Zeugophora annulata

XML Treatment for Zeugophora chujoi

XML Treatment for Zeugophora flavonotata

XML Treatment for Zeugophora nigricolis

XML Treatment for Zeugophora unifasciata

XML Treatment for Zeugophora varipes

XML Treatment for Zeugophora hozumii

XML Treatment for Zeugophora japonica

XML Treatment for Zeugophora cupka

XML Treatment for Zeugophora gracilis

XML Treatment for Phyllotreta ezoensis

XML Treatment for Phyllotreta shirahatai

XML Treatment for Longitarsus aff. holsaticus

XML Treatment for Dibolia japonica

XML Treatment for Mantura clavareaui

XML Treatment for Mantura fulvipes

XML Treatment for Mantura japonica

XML Treatment for Hippuriphila babai

XML Treatment for Psylliodes punctifrons

XML Treatment for Psylliodes aff. subrugosa

XML Treatment for Halticorcus kasuga

XML Treatment for Halticorcus sauteri

XML Treatment for Halticorcus hiranoi

XML Treatment for Halticorcus duodecimmaculata

XML Treatment for Argopistes coccinelliformis

XML Treatment for Argopistes biplagiata

XML Treatment for Argopistes tsekooni

XML Treatment for Argopistes ryukyuensis

XML Treatment for Argopistes unicolor

XML Treatment for Argopus balyi

XML Treatment for Argopus clarki

XML Treatment for Argopus clypeatus

XML Treatment for Argopus punctipennis

XML Treatment for Argopus nigripennis

XML Treatment for Argopus unicolor

XML Treatment for Sphaeroderma nigricolle

XML Treatment for Sphaeroderma japanum

XML Treatment for Sphaeroderma tarsatum

XML Treatment for Sphaeroderma seriatum

XML Treatment for Sphaeroderma apicale

XML Treatment for Sphaeroderma akebia

XML Treatment for Sphaeroderma inaizumii

XML Treatment for Sphaeroderma quadrimaculatum

XML Treatment for Sphaeroderma flavonotatum

XML Treatment for Sphaeroderma separatum

XML Treatment for Sphaeroderma placidum

XML Treatment for Sphaeroderma unicolor

XML Treatment for Sphaeroderma uenoi

XML Treatment for Sphaeroderma ohkuboi

XML Treatment for Sphaeroderma balyi

XML Treatment for Sphaeroderma fulvoapicale

XML Treatment for Sphaeroderma komiana

XML Treatment for Leptispa taguchii

XML Treatment for Leptispa miyamotoi

XML Treatment for Asamangulia yonakuni

XML Treatment for Dactylispa subquadrata

XML Treatment for Dactylispa masonii

XML Treatment for Dactylispa angulosa

XML Treatment for Dactylispa higoniae

XML Treatment for Dactylispa adinae

XML Treatment for Dactylispa issikii

XML Treatment for Hispellinus moerens

XML Treatment for Rhadinosa nigrocyanea

XML Treatment for Platypria melli

XML Treatment for Dicladispa boutani

XML Treatment for Notosacantha ihai

XML Treatment for Notosacantha loochooana

XML Treatment for Notosacantha nishiyamai

## ﻿Additional information

### Author ORCIDs

Yume Imada https://orcid.org/0000-0003-2173-7389

### Data availability

All of the data that support the findings of this study are available in the main text.
